# A single liver-sparing myotropic AAV ameliorates muscular dystrophy in humanized mice by base editing

**DOI:** 10.1172/JCI202378

**Published:** 2026-11-02

**Authors:** Yu-Chung Pien, Hui Li, Gabrielle Bulliard, Huachun Liu, Mateusz Z. Durbacz, Zhisheng Xu, Andreas C. Chai, Francesco Chemello, Efrain Sanchez-Ortiz, Nerea Zabaleta, Luk H. Vandenberghe, Ning Liu, Eric N. Olson

**Affiliations:** 1 Department of Molecular Biology,; 2 Hamon Center for Regenerative Science and Medicine, and; 3 Senator Paul D. Wellstone Muscular Dystrophy Specialized Research Center, University of Texas Southwestern Medical Center, Dallas, Texas, USA.; 4 Grousbeck Gene Therapy Center, Schepens Eye Research Institute, and Massachusetts Eye and Ear Infirmary, Harvard Medical School, Boston, Massachusetts, USA.; 5 The Broad Institute of Harvard and MIT, Cambridge, Massachusetts, USA.; 6 Cima Universidad de Navarra, Pamplona, Spain.

**Keywords:** Genetics, Muscle biology, Skeletal muscle

## Abstract

Duchenne muscular dystrophy (DMD) is caused by mutations in the *DMD* gene, most of which are large exon deletions that result in loss of dystrophin protein. Adenine base editing (ABE) enables permanent gene correction by modifying splice sites to cause exon skipping and restoration of the *DMD* open reading frame. We previously demonstrated that adeno-associated virus 9–mediated (AAV9-mediated) delivery of adenine base editors allows efficient gene editing in mouse models of DMD. However, conventional AAV-based gene delivery approaches are complicated by their limited packaging capacity and high-dose-related toxicity. Here, we describe a single AAV vector encoding a compact adenine base editor, SlugABE8e, and a single-guide RNA, which enable efficient editing in a humanized DMD mouse model at a lower dose using the muscle-tropic MyoAAV4 capsid. Systemic MyoAAV4A delivery in juvenile DMD mice efficiently restored dystrophin expression in the heart and skeletal muscles. This molecular rescue was accompanied by reduced serum creatine kinase activity and diminished muscle pathology. A liver-detargeted MyoAAV4E-off capsid eliminated viral entry and editing in the liver. We also explored a high-fidelity base editor, SlugHF-ABE8e(V106W), which showed similar on-target and off-target editing to SlugABE8e. Together, these findings establish an all-in-one AAV strategy that efficiently mitigates DMD disease progression.

## Abstract 2

A single, muscle-targeted gene editing AAV restores dystrophin and improves muscle function in humanized Duchenne muscular dystrophy mice while bypassing the liver.

## Introduction

Duchenne muscular dystrophy (DMD) is characterized by fatal cardiac and skeletal muscle degeneration. DMD is caused by mutations in the X-linked *DMD* gene, encoding dystrophin, one of the largest proteins in the human genome (1). Dystrophin serves as a linker between the actin cytoskeleton and the extracellular matrix, thereby preventing muscle damage during contraction (2, 3). Large exon deletions in the *DMD* gene, which are present in >70% of the DMD patient population (4), often lead to frame shifts in the coding sequence, thereby preventing translation of the essential carboxy terminus of dystrophin. Several mutational hotspots have been identified within the *DMD* gene, including exons 6–7, exons 43–46, and exons 50–53 (5). Therapeutic strategies that enable skipping of out-of-frame exons allow restoration of the *DMD* open reading frame and expression of truncated but functional forms of dystrophin, due to the partial redundancy of the rod domains, encoded by exons 9–61 (6).

Adenine base editing (ABE), which performs A•T to G•C conversion, is mediated by a base editor, comprising an engineered adenine deaminase domain fused to a Cas9 nickase, and a single guide RNA (sgRNA). Base pairing of the sgRNA with complementary genomic sequences allows for editing of target bases within a base-editing window when a protospacer adjacent motif (PAM) sequence is also present. ABE enables *DMD* exon skipping by changing the canonical splice acceptor site (SAS) “ag” to “gg.”

Adeno-associated viruses (AAVs) are widely used delivery vehicles for in vivo gene editing given their versatile tissue tropisms. For example, AAV9 displays preferential tropism for muscles and the heart (7), but it also accumulates in the liver, causing severe hepatotoxicity. Owing to the packaging limit of AAVs (4.8 kb) and the large size of ABE components, dual-AAV9s with a split-intein-mediated trans-splicing system have been used to reconstitute base editors in vivo (8). Previous studies showed efficient base editing and disease correction in DMD mice using SpCas9-ABE8e and SpCas9-ABEmax by dual-AAV9s (8, 9).

While dual-AAV9s encoding base editors have shown promising results in mice, their clinical translation is hindered by scalability, inefficient and truncated expression of base editors, and high-dose-related toxicity (10–12). Because muscle tissues constitute up to 40% of the human body mass, the high doses of AAV9 required for muscle transduction can cause serious liver toxicity (10, 11). High doses of AAV have also induced cardiogenic shock in rare cases (12). These adverse events highlight the need for alternative capsids with higher transduction efficiency and decreased toxicity. There is also a critical need to identify smaller base editors that can be packaged into single AAV vectors and for further capsid optimization.

*Streptococcus pyogenes* Cas9 (SpCas9), the most widely used Cas9 homolog, is 1,368 amino acids (aa) and exhibits robust editing efficiency (13). Several smaller Cas9 homologs have been identified with more restricted PAM recognition sequences compared with SpCas9 [-NGG], including SaCas9 (1,053 aa) [-NNGRRT], Nme2Cas9 (1,082 aa) [-NNNNCC], and SlugCas9 (1,055 aa) [-NNGG] (14–17). Engineered forms of Cas9, such as SaKKH-Cas9 [-NNHRRT] from SaCas9 [-NNGRRT] (18), can broaden the targeting scope with a more flexible PAM. Moreover, ABE is compatible with smaller Cas9 variants (19). The reduced size of base editors facilitates their packaging into a single AAV vector for therapeutic application (20).

Myotropic AAVs are a class of modified AAVs that carry an integrin-binding 7-mer RGD peptide in the variable region VIII (VR-VIII) of the capsid (21). Two variants, MyoAAV4A and MyoAAV4E, showed superior transduction to skeletal and cardiac muscles compared with AAV9 in nonhuman primates (22). To further enhance the specificity of transduction and limit toxicities related to vector biodistribution to the liver, G267A and S269T mutations were introduced in the VR-1 variable region, resulting in the engineered MyoAAV4E-GAST that was shown to substantially diminish liver targeting while maintaining enhanced muscle uptake in mice compared with AAV9 (23, 24). These advances increased the compatibility and safety profile of single AAV base editing for precise DMD gene correction.

In this study, we took advantage of the compact size, targeting flexibility, and higher editing activity of several miniature adenine base editors. By screening in HEK293T cells, we identified high base-editing efficiency of Slug-ABE8e for the *DMD* exon 45 SAS. All-in-one AAV-SlugABE8e restored dystrophin expression in patient-derived induced pluripotent stem cell cardiomyocytes (iPSC-CMs) and a humanized DMD mouse model. MyoAAV4E-off eliminated liver AAV uptake, and the high-fidelity SlugHF-ABE8e(V106W) variant showed a similar gene editing profile compared with Slug-ABE8e. Our findings highlight the therapeutic potential of all-in-one base editing using myotropic capsids with a lower dose and toxicity in treating DMD.

## Results

### Editing of therapeutically relevant DMD exons with compact adenine base editors.

Mutations in *DMD* exons 43–53 were amenable to exon skipping, allowing production of a truncated yet functional dystrophin protein (Supplemental Figure 1; supplemental material available online with this article; https://doi.org/10.1172/JCI202378DS1) (25). Exon skipping within this mutational hotspot could potentially benefit approximately 47% of patients with DMD (Figure 1A).

We designed ABE exon-skipping strategies targeting the canonical SAS at the 5’ ends of target exons by changing the nucleotides from “ag” to “gg” (Figure 1B). To identify compact ABEs, we started by screening a panel of smaller Cas9 variants (Supplemental Table 1). On average, these Cas9 variants are approximately 300 amino acids smaller than SpCas9 (1,368 aa), making them suitable for single AAV vector packaging.

SaCas9, SaKKH-Cas9, Nme2Cas9, and SlugCas9 were selected based on their flexible PAM requirements within *DMD* exons (15–18). These Cas9 variants were engineered with ABE8e, an evolved TadA variant compatible with non-SpCas9 variants owing to its optimized deamination kinetics (19). Several strategies were implemented to enhance base-editing efficiency and expand the targeting range. Domain-inlaid Nme2-ABE8e variants shifted the editing window and improved editing efficiency (26), while a structure-guided PAM-interacting domain switch alleviated the PAM restriction of Nme2^smu^-ABE8e-i1, Nme2^smu^-ABE8e-i8, and Nme2^smu^-ABE8e-i1^E932R^ [-NNNNCN] (26). In addition, phage-assisted evolution of eNme2.T-ABE8e and eNme2.C-ABE8e [-NNNNCN, -NNNNTN] and Slug-ABE8e [-NNGN] further relaxed PAM requirements (Figure 1C) (27, 28).

We conducted a screen using 9 compact ABEs across 87 ABE-sgRNA combinations targeting *DMD* exons 44, 45, 50, 51, 52, and 53 in HEK293T cells (Figure 1, C and D). sgRNAs were selected based on the optimal editing window of each mini-ABE (Supplemental Figure 2, A–G). SaCas9 and SlugCas9 shared the same SaCas9 sgRNA scaffold. To avoid polyT stretch-induced premature transcriptional termination by the RNA III polymerase promoter, we used a modified SaCas9 sgRNA scaffold (Supplemental Figure 3) (29).

Following transient expression of mini-ABEs and their corresponding sgRNAs, A-to-G nucleotide editing of the SAS was evaluated by Sanger sequencing. In exons 50, 51, and 53, Nme2^Smu^-i1^E932R^-ABE8e exhibited the highest editing efficiencies (15%–30%) among all mini-ABEs (Figure 2A). The generally lower editing efficiency in these exons may be attributed to the unavoidable polyT stretches in the spacer sequences (Supplemental Figure 2, C, D, and F). In exons 44 and 52, eNme2.C-ABE8e and Nme2^Smu^-i1^E932R^-ABE8e achieved up to 50% editing efficiency (Figure 2B). Notably, Slug-ABE8e reached 60% A-to-G editing of the SAS of exon 45, comparable to the previously reported Sp-ABE8e and sgRNA targeting the same exon 45 (Figure 2C) (8).

The best performing mini-ABEs for each exon were then transiently transfected into WT hiPSC-CMs (Figure 2D). Comparable editing levels (~30%) were observed for exons 45, 50, 51, 52, and 53, with lower editing (10%) in exon 44 (Figure 2E). The difference in editing efficiency between HEK293T cells and hiPSC-CMs likely reflects differences in transfection efficiency and chromatin accessibility between these cell types (30).

### Design of an all-in-one AAV delivery method for ABE correction of DMD.

AAV is a widely used gene-delivery vector in clinical trials and is approved by the US FDA (31). The AAV packaging limit is approximately 4.8 kb. In our all-in-one ABE constructs, we included the essential components for *DMD* gene editing: a muscle-specific CK8e promoter (~0.43 kb), a short synthetic poly(A) signal (~0.06 kb), and a U6 promoter-sgRNA cassette (~0.36 kb). We omitted the approximate 0.25 kb woodchuck hepatitis virus posttranscriptional regulatory element 3 (WPRE3), which was used in previous dual-AAV designs for enhanced transgene expression (Supplemental Figure 4A) (8).

All-in-one constructs encoding Slug-ABE8e and essential elements fit within the 4.8 kb limit, whereas eNme2.C-ABE8e and Nme2^Smu^-i1^E932R^-ABE8e exceeded this size (Supplemental Figure 4B). To package Nme2-ABE variants within a single AAV, we substituted the CK8e promoter with a ubiquitous EFS promoter (~0.25 kb) and the U6 promoter with a miniU6 (~0.1 kb) promoter. However, the ubiquitous expression of Nme2-ABE raises concerns about nonmuscle editing, and the lower transcriptional activity of miniU6 may reduce sgRNA expression and the editing efficiency (32). Therefore, we selected Slug-ABE8e, driven by the CK8e promoter, and an exon 45-targeting sgRNA for muscle-specific targeting.

### ABE correction of DMD patient–derived hiPSC-CMs.

To validate the therapeutic potential of the all-in-one AAV ABE system, we tested it in DMD patient–derived iPSC-CMs harboring an exon 44 deletion (ΔEx44). In the all-in-one Slug-ABE8e construct, the base editor and sgRNA cassettes were arranged in tandem. We packaged the base-editing components into an AAV6 capsid, an AAV variant with high transduction efficiency in hiPSC-CMs (Figure 3A) (33). Three weeks after AAV6 delivery in ΔEx44 hiPSC-CMs, Slug-ABE8e achieved 76.3% ± 9.5% on-target A12 editing at the genomic level (Figure 3, B and C). Although some bystander editing occurred, it was functionally inconsequential due to the exon skipping strategy (Supplemental Figure 5).

RT-PCR analysis revealed a shift in cDNA band size (Figure 3D) (34). Skipping exon 45 in ΔEx44 hiPSC-CMs resulted in 37.0% ± 4.1% restoration of dystrophin protein, as confirmed by both Western blotting and immunostaining (Figure 3, E–G), demonstrating the effectiveness of the all-in-one AAV SlugABE system.

### Systemic myotropic-AAV delivery restores dystrophin expression in heart and skeletal muscle in juvenile ΔEx44;hEx45 DMD mice.

Systemic administration of AAV9-ABEs in neonatal DMD mice has previously been shown to restore dystrophin expression via exon skipping and to prevent pathological disease progression (8). However, current dual-AAV doses used in in vivo DMD studies remain relatively high (1 × 10^14^ vg/kg to 3 × 10^14^ vg/kg), posing a challenge for clinical translation (8, 35, 36). To address this, we evaluated systemic delivery of an all-in-one AAV base editing-system in juvenile DMD mice using a lower virus dose.

Previously, we generated a humanized ΔEx44;hEx45 DMD mouse model by deleting DMD exon 44 and replacing mouse exon 45 with its human homolog (37). These mice lack dystrophin protein and displayed reduced forelimb grip strength and increased serum creatine kinase (CK) activity (Supplemental Figure 6, A–D). ΔEx44;hEx45 DMD mice did not exhibit detectable cardiac dysfunction by fractional shortening score compared with WT mice at 8 weeks (Supplemental Figure 6E). We selected 2 engineered capsids, MyoAAV4A and MyoAAV4E, for their cardiac and skeletal muscle transduction in vivo (22). The MyoAAV4E capsid was further engineered into a liver detargeted variant, MyoAAV4E-GAST (24), owing to its greater transduction in nonhuman primates than MyoAAV4A (22). Hereafter, MyoAAV4E-GAST will be referred to as MyoAAV4E-off.

We tested 2 cargo designs in which the base editor and sgRNA cassettes were arranged either in tandem (cargo 1) or in an inverted configuration (cargo 2). Cargo 1 was packaged into MyoAAV4A, and cargo 2 into MyoAAV4E-off (Figure 4A).

We administered MyoAAV4A-Slug-ABE8e and MyoAAV4E-off-SlugABE8e via retro-orbital injection into 4-week-old ΔEx44;hEx45 DMD mice at a dose of 5 × 10^13^ vg/kg (Figure 4B). Four weeks after injection, the heart, diaphragm, tibialis anterior (TA), triceps, quadriceps, gastrocnemius-plantaris (GP), soleus, and extensor digitorum longus (EDL) were collected for analysis. MyoAAV4A achieved an A-to-G editing efficiency of 20.2% ± 3.2% (heart), 4.6% ± 2.3% (diaphragm), 6.0% ± 1.9% (TA), 8.4% ± 2.7% (triceps), 9.3% ± 1.8% (quadriceps), 7.2% ± 2.6% (GP muscle), 7.2% ± 3.1% (soleus), and 10.3% ± 4.0% (EDL), while MyoAAV4E-off achieved similar editing efficiency of 16.0% ± 2.6%, 4.9% ± 2.5%, 9.2% ± 3.7%, 10.2% ± 3.7%, 6.1% ± 2.6%, 3.3% ± 2.2%, 7.1% ± 1.8%, and 8.1% ± 4.5%, respectively, for these tissues (Figure 4C). Editing efficiencies at the DNA level are likely underestimated, given the myocyte-specific expression of ABEs and the inclusion of unedited nonmuscle cells such as endothelial cells and immune cells in tissue samples (38, 39). To more accurately assess exon skipping, RT-PCR and deep amplicon sequencing were performed on *DMD* transcripts.

MyoAAV4A achieved exon 45 skipping efficiencies of 75.9% ± 11.3% (heart), 33.8% ± 8.6% (diaphragm), 35.8% ± 4.1% (TA), 37.1% ± 9.7% (triceps), 29.2% ± 9.2% (quadriceps), 31.8% ± 7.5% (GP), 31.8% ± 10.9% (soleus), and 35.9% ± 9.9% (EDL), while MyoAAV4E-off achieved similar efficiency of 61.8% ± 12.2%, 24.4% ± 11.4%, 35.5% ± 9.4%, 40.5% ± 5.5%, 26.4% ± 8.3%, 24.7% ± 8.1%, 41.3% ± 9.7%, and 30.4% ± 13.1%, respectively, in these tissues (Figure 4D).

Western blot analysis showed dystrophin expression achieving 80.5% ± 25.2% (MyoAAV4A) and 72.3% ± 41.6% (MyoAAV4E-off) of WT levels in the heart (Figure 4, E and G). In skeletal muscles, dystrophin restoration was lower: 13.0% ± 3.7% and 9.3% ± 6.8% (diaphragm), 19.6% ± 6.3% and 21.3% ± 8.8% (TA), and 13.8% ± 8.3% and 13.5% ± 6.2% (triceps), by MyoAAV4A and MyoAAV4E-off, respectively (Figure 4, F and G).

We examined viral genome copies of the 2 capsids. MyoAAV4A had higher viral genome distribution in the heart and slightly lower in the muscles compared with MyoAAV4E-off (Supplemental Figure 7). We further assessed the cargo expression by the 2 capsids. Base editor and sgRNA expression in MyoAAV4A-injected mice were 4.6 and 4.1-fold (heart), 1.3 and 1.4-fold (diaphragm), 2.0 and 1.0-fold (TA), 1.3 and 0.4-fold (triceps), 5.3 and 4.6-fold (quadriceps), and 3.1 and 1.0-fold (GP), respectively, over MyoAAV4E-off-injected mice (Supplemental Figure 8, A–F). These results correspond to the previously reported transduction profiles by the 2 capsids in mice (22).

Although the 2 capsids achieved comparable levels of dystrophin restoration in vivo, MyoAAV4E-off markedly reduced liver transduction, lowering viral genome copies to baseline, whereas MyoAAV4A produced approximately 110 DNA vector copies per diploid genome (Figure 4H). Accordingly, despite using a muscle-specific promoter, A-to-G editing of the target site reached 0.5% ± 0.5% at the DNA level in the liver of MyoAAV4A-injected mice but was undetectable in MyoAAV4E-off–treated animals (Figure 4I). Together, these results demonstrate efficient muscle DNA editing and depletion of liver viral entry using liver detargeting muscle-tropic AAVs for DMD correction.

### Improvement of muscle integrity and function after systemic all-in-one AAV treatment.

We next assessed pathological muscle features in ΔEx44;hEx45 DMD mice following systemic AAV treatment. Muscle tissues from untreated 4-week-old mice and 8-week-old mice treated at 4 weeks with saline or AAVs were analyzed histologically. H&E staining of diaphragm, TA, and triceps revealed myofiber disorganization, centralized nuclei, and fibrosis in 4-week-old ΔEx44;hEx45 DMD mice (Figure 5A) — typical features of chronic muscle degeneration and regeneration. After 4 weeks, saline-treated mice displayed worsened pathology, including increased fibrosis and calcification, whereas MyoAAV4A and MyoAAV4E-off treatment ameliorated these disease features (Figure 5A and Supplemental Figure 9). In the TA muscle, ΔEx44;hEx45 DMD mice exhibited 61.2% ± 8.2% centrally nucleated fibers. Treatment with MyoAAV4A and MyoAAV4E-off significantly reduced the percentage of centrally nucleated fibers to 44.7% ± 3.5% and 46.5% ± 4.2%, respectively, indicating that dystrophin restoration partially relocalized myonuclear position and improved muscle histopathology (Figure 5B). Picrosirius red staining showed 31.5% ± 4.8% of fibrosis in ΔEx44;hEx45 DMD diaphragm, and neither MyoAAV4A (26.7% ± 3.8%) nor MyoAAV4E-off (27.1% ± 5.9%) treatment significantly reduced fibrosis. TA muscle from ΔEx44;hEx45 DMD mice showed 21.0% ± 5.2% of fibrosis, which was significantly reduced following treatment with MyoAAV4A (13.4% ± 2.8%) and MyoAAV4E-off (13.5% ± 1.6%) (Figure 5, C and D). These findings suggest that AAV treatment effectively reduced fibrosis in moderately affected muscle such as TA but was insufficient to reverse the more advanced fibrosis present in the diaphragm. No fibrosis was observed in the hearts of AAV-injected and saline-injected ΔEx44;hEx45 DMD mice (Figure 5D and Supplemental Figure 10, A and B).

Dystrophin immunostaining confirmed nearly complete dystrophin restoration in hearts of both AAV-treated groups. In skeletal muscles (diaphragm, TA, and triceps), dystrophin expression was patchy, with dystrophin-negative regions corresponding to damaged fibers (Figure 5, A and E, and Supplemental Figure 10C). Myofiber cross-sectional area distributions showed a higher proportion of small myofibers in saline-treated ΔEx44;hEx45 DMD mice than WT mice, with MyoAAV4A and MyoAAV4E-off treatments showing a modest trend toward larger myofiber areas (Figure 5F and Supplemental Figure 11A). Mean myofiber cross-sectional area was lower in saline-treated ΔEx44;hEx45 DMD mice (2,086.3 ± 123.8 μm^2^) than in WT mice (3,671.8 ± 322.1 μm^2^). Both MyoAAV4A (2,436.8 ± 578.2 μm^2^) and MyoAAV4E-off (2,435.3 ± 459.4 μm^2^) treatments showed higher myofiber cross-sectional areas than untreated DMD mice, though these differences did not reach statistical significance (Supplemental Figure 11B). Overall, these data indicated that MyoAAV4 treatments partially improved muscle integrity.

Functionally, ΔEx44;hEx45 DMD mice exhibited reduced forelimb grip strength at 4 weeks (Supplemental Figure 6B). At 8 weeks, saline-treated mice exhibited significantly lowered strength (2.5 ± 0.3 gram-force/body weight [gf/g]) compared with that of WT controls (4.8 ± 0.3 gf/g). MyoAAV4A-treated mice showed a 28.4% improvement in grip strength (3.8 ± 0.5 gf/g), while MyoAAV4E-off treatment produced a modest 11.7% increase (3.0 ± 0.6 gf/g) (Figure 6A). In addition, the remaining grip strength showed enhanced grip strength durability in MyoAAV4A and MyoAAV4E-off–treated mice compared with that of saline-treated DMD mice (Figure 6B). We also performed a downhill treadmill exercise experiment after 4 weeks of AAV treatments without pretraining. ΔEx44;hEx45 DMD mice showed markedly reduced exercise endurance at 19 minutes compared with that of WT mice at 52 minutes. MyoAAV4A significantly improved exercise endurance at 42 minutes compared with DMD mice, whereas MyoAAV4E-off showed a similar trend at 26 minutes that did not reach statistical significance (Figure 6C). These data indicated that both MyoAAV4 treatments partially restored muscle strength and exercise endurance.

We next quantified activity of serum CK, a sensitive marker of muscle injury. Four-week-old ΔEx44;hEx45 DMD mice exhibited 9.3-fold higher serum CK levels than WT controls under sedentary conditions. Following grip strength analyses, CK activity increased approximately 133-fold in ΔEx44;hEx45 DMD mice relative to that in WT mice (Supplemental Figure 6C). Four weeks after treatment, saline-treated mice retained high serum CK (~100-fold), while MyoAAV4A and MyoAAV4E-off mice showed substantial reductions (4.8-fold and 7.8-fold, respectively) (Figure 6D), indicating attenuated muscle damage.

### No liver toxicity or induced immunogenicity was observed after systemic all-in-one AAV treatment.

To examine potential liver toxicity by AAV administration, we detected serum alanine aminotransferase (ALT), aspartate aminotransferase (AST), alkaline phosphatase (ALKP), and total bilirubin (TBIL) levels as indicators of potential liver injury (Figure 6E). ALT and AST were elevated in our humanized DMD mice compared with WT mice (Figure 6E), probably due to the ongoing muscle damage that likely contributed to high ALT and AST activity in the liver. Both enzyme activities were reduced after MyoAAV4A and MyoAAV4E-off treatments. To better evaluate potential liver toxicity, we included serum ALKP and TBIL as additional safety biomarkers, as neither is expressed in skeletal muscle. Serum analyses showed no differences in either ALKP or TBIL activity among all groups, indicating no obvious liver damage (Figure 6E). We further investigated **γ**-glutamyl transferase (GGT), which is widely used as a marker for liver toxicity in clinical assessment. GGT activity was below our detection limit (data not shown).

Since AAV administration can induce both innate and adaptive immune responses and bacterially derived base editors can trigger T cell responses, we investigated the immunogenicity induced by AAV treatment (40). We performed qPCR analysis of the cellular immune response markers *Tnf* and *Ifng* to evaluate potential immunogenicity of the SlugABE8e in the liver. *Tnf* is an indicator of the innate immune response, and *Ifng* is an indicator of the T cell immune response. We further examined the heart and TA muscle, where SlugABE8e was expressed. We did not observe any elevation of *Tnf* or *Ifng* in the heart, TA muscle, or liver after AAV administration, suggesting that SlugABE8e delivery did not elicit detectable local inflammatory responses under our experimental conditions (Figure 6F).

### Analysis of off-target DNA editing in DMD ΔEx44 hiPSC-CMs.

Despite the high efficiency of base editing, off-target mutations remain a safety concern (41). To enhance editing specificity, numerous strategies have been deployed. We engineered Slug-ABE8e into a high-fidelity variant, SlugHF-ABE8e(V106W), combining a high-fidelity nSlugCas9 (SlugHF) and an ABE8e(V106W) variant with reduced RNA off-target (17,19). The SlugCas9-HF mutations reduce polar interactions with DNA, thereby increasing targeting specificity (17), while the ABE8e-V106W minimized RNA off-target editing by reducing the tRNA binding pocket through mutating valine into a larger tryptophan (42).

We packaged both Slug-ABE8e and SlugHF-ABE8e(V106W) into AAV6 and treated ΔEx44 hiPSC-CMs with different viral doses. This high-fidelity variant showed lower on-target editing compared with Slug-ABE8e (Figure 7, A and B). Deep amplicon sequencing of *DMD* transcripts showed 85.0% ± 3.6% exon skipping (Supplemental Figure 12A) and 3.0% ± 0.7% cryptic splice site deletions in 1 × 10^5^ vg/cell AAV6-SlugHF-ABE8e(V106W)–treated ΔEx44 hiPSC-CMs (Supplemental Figure 12, B and C). Western blot and immunostaining confirmed complete dystrophin restoration (Supplemental Figure 12, D–F), demonstrating robust on-target editing by SlugHF-ABE8e(V106W).

To assess potential off-target DNA editing, Cas-OFFinder was used to predict candidate loci with less than or equal to 4 mismatches in the spacer sequence. Given that SaCas9 tolerates 1–2 mismatches in the PAM-distal region (18), we prioritized sites with similar mismatch patterns (Figure 7C). All 20 candidate loci were located in noncoding, intronic, or intergenic regions. We also compared Slug-ABE8e with the SlugHF-ABE8e(V106W). Because SlugHF-ABE8e(V106W) showed lower on-target editing than Slug-ABE8e at the same AAV dose (Figure 7, A and B), we selected viral doses that achieved comparable on-target editing efficiencies before evaluating off-target activity (Figure 7B). Deep sequencing detected no measurable editing at 16 of the 20 predicted off-target sites. Low levels of A-to-G editing (1%–9%) were detected at 4 intronic or intergenic sites (sites 1 [intronic], 4 [intergenic], 5 [intronic], and 7 [intronic]), with SlugHF-ABE8e(V106W) showing slightly lower editing than Slug-ABE8e at these sites (Figure 7D). The biological consequences of these rare events remain to be investigated. Notably, the mismatches at these three 4-mismatch loci (sites 4, 5, and 7) were predominantly located in the sgRNA-distal region (N11-N21 in Figure 7C), where the mismatches at site 1 were closer to the PAM region. This pattern is consistent with the well-established positional effect of Cas9 target recognition, in which mismatches distal to PAM are generally better tolerated than those within the PAM-proximal seed region (43), suggesting that the position of mismatches, particularly in the sgRNA-distal region, is a more important determinant of off-target editing activity than mismatch number alone.

Taken together, these results demonstrate that our all-in-one AAV system mediates efficient and specific base editing, enabling *DMD* exon skipping and restoration of dystrophin expression, while minimizing off-target effects and pathological remodeling.

## Discussion

In this study, we engineered a compact base editor and a single-AAV ABE system for efficient correction of DMD mutations. Among all miniature base editors tested, Slug-ABE8e showed the highest editing efficiency at the *DMD* exon 45 SAS. In ΔEx44 hiPSC-CMs, AAV6-Slug-ABE8e enabled approximately 75% on-target editing and restored approximately 37% dystrophin protein expression. In ΔEx44;hEx45 DMD mice at P28, MyoAAV4A and MyoAAV4E-off delivery of Slug-ABE8e resulted in 72%–80% and 9%–21% dystrophin protein restoration in the heart and skeletal muscles, respectively, using doses 3 times lower than previously reported (8). SAS editing led to improved muscle integrity, grip strength, muscle endurance, and reduced serum CK activity. Notably, MyoAAV4E-off avoided liver transduction, as demonstrated by reduced viral entry and limited nonmuscle editing. The SlugABE8e and SlugHF-ABE8e(V106W) variants exhibited high on-target editing with minimal off-target editing in ΔEx44 hiPSC-CMs.

Our all-in-one base editing strategy integrates several key advances in gene editing for DMD. First, we engineered a miniABE, Slug-ABE8e, with comparable activity and similar PAM requirement to SpCas9-ABE8e (28). Second, we designed an all-in-one AAV vector system that circumvents the limitations of dual-AAV approaches, which rely on intein-mediated reconstitution of full-length editors, often producing truncated proteins and requiring higher doses. Third, we employed a next-generation AAV capsid, MyoAAV4E-off, optimized for muscle and cardiac tropism and reduced liver transduction. These approaches enabled a 2- to 6-fold reduction in AAV dose while maintaining robust editing efficiency. Moreover, we performed base editing after disease onset in humanized ΔEx44;hEx45 DMD mice at P28, whereas most prior studies used mice at P14 or earlier (8, 9, 35). Finally, we engineered a high-fidelity variant, SlugHF-ABE8e(V106W), which maintained high DNA on-target activity and potentially reduced DNA and RNA off-target editing.

Approximately 8% of patients with DMD could benefit from exon 45 skipping to restore the *DMD* open reading frame, including those carrying exon 44 deletions (5). Although full-length dystrophin restoration was not achieved, previous estimates suggest that as little as 15% dystrophin expression can result in a milder Becker muscular dystrophy phenotype, and 30% restoration may prevent skeletal muscle symptoms (44). Our strategy showed early functional improvement in ΔEx44;hEx45 DMD mice at 4 weeks after treatment. Furthermore, an extended period after treatment may allow for greater functional and histological improvement in skeletal muscle. Notably, the mechanisms underlying the superior muscle function outcomes observed with MyoAAV4A compared with MyoAAV4E-off remain to be elucidated. Since ΔEx44;hEx45 DMD mice did not exhibit heart failure that recapitulated that of patients with DMD under our experimental conditions, long-term assessments, when cardiac dysfunction becomes evident in this model (approximately 8–10 months of age), will be necessary to address physiological benefit in the heart.

Microdystrophin-based gene therapy has advanced to several clinical trials (ClinicalTrials.gov; identifier: NCT05693142, NCT06138639, and NCT06270719). However, the phase III EMBARK trial of Sarepta’s *Delandistrogene moxeparvovec* (Elevidys) did not demonstrate a significant improvement in North Star Ambulatory Assessment scores (45, 46), and some patients developed an immune response to microdystrophin. In contrast, CRISPR/Cas9 nuclease-based gene editing offers the possibility of permanent exon reframing or exon skipping of the *DMD* gene by nonhomologous end joining to restore dystrophin expression (47, 48). However, DNA double-stranded breaks can induce genotoxic byproducts, which raise concerns for clinical translation (49). Base editing provides a potentially safer alternative, allowing precise nucleotide conversion without double-stranded breaks (50). The ABE-mediated exon 45–skipped ΔEx44 dystrophin product (~3,360 aa) more closely resembles the full-length dystrophin (3,684 aa) than microdystrophins such as Elevidys (1,325 aa) (Supplemental Figure 13). Although split-intein systems can deliver full-length dystrophin via 3 AAVs, they face challenges in manufacturing complexity, truncated protein products, and dose-related toxicity (51, 52).

Although immune responses to Cas9-based genome editors have been reported, we did not comprehensively evaluate a potential SlugABE8e-induced immune response in ΔEx44;hEx45 DMD mice. A comprehensive evaluation of the immunogenicity of SlugCas9, including direct comparison with more widely used Cas9 orthologs such as SpCas9, will be important for future preclinical studies. Strategies such as epitope engineering of SaCas9 have mitigated immunogenicity in animal models (40, 53, 54). Similar approaches could be applied to SlugCas9 to reduce immunogenicity in patients. Further characterization of immune cell infiltration or longer-term assessment of potential immune-mediated clearance will also be necessary to define the immune responses to AAV administration.

We did not explore all possible compact ABE options for *DMD* exon skipping, as splicing donor sites were not included in our screen. Other small Cas9 variants with compatible PAMs, such as SauriCas9 [-NNGG], may offer additional opportunities. Although Slug-ABEs showed robust editing both in vitro and in vivo, lower SlugCas9 expression compared with other small Cas9 variants suggests reduced protein stability (43). Future protein engineering could enhance SlugCas9 stability and performance. Moreover, emerging AI-guided protein design and few-shot learning approaches may accelerate the directed evolution of both Cas9 and adenine deaminase domains to improve editing efficiency and further reduce the required AAV dose (55, 56).

High-dose AAV administration can cause life-threatening hepatotoxicity (57), as natural AAVs tend to accumulate in the liver. Mutations in the VR-I region in AAV have been shown to reduce hepatic tropism (23, 58, 59). The potential of systemic AAV-associated toxicity, including cytokine storm, capillary leak syndrome, complement-mediated thrombotic microangiopathy, acute respiratory distress syndrome, and cardiogenic shock, highlights the clinical need for lower AAV doses (12, 60–62). Our MyoAAV4E-off vector achieved high editing efficiency at reduced doses while avoiding liver targeting. We did not observe liver damage or immune responses by MyoAAV4 in the liver, heart, or muscles in ΔEx44;hEx45 DMD mice. We did not assess potential cardiac injury by measuring serum troponin levels following administration of MyoAAV4 capsids. Future studies in other species, such as nonhuman primates, will be necessary to further evaluate the potential benefits of the MyoAAV4E-off vector in mitigating liver toxicity. Muscle degeneration was reversed by dystrophin restoration, as evidenced by reduced CK activity. Although dystrophin restoration reduced fibrosis in TA muscle, it did not reverse severe fibrosis in the diaphragm. Further quantitative analysis across different muscle tissues, including the percentage of dystrophin-positive fibers and the cross-sectional area and centralized nuclei of dystrophin-positive and dystrophin-negative fibers, will provide a more comprehensive assessment of the therapeutic benefit, particularly in the diaphragm. Overall, we observed prevention of further worsening of the phenotypes, underscoring the importance of early intervention. Fibrosis reduces AAV uptake (63), and engineering fibrosis-resistant AAV variants could improve transduction efficiency in advanced disease stages. Additionally, preexisting anti-AAV antibodies limit treatment in patients; the IgG-specific protease imlifidase offers a promising strategy to degrade such antibodies and enable AAV redosing (64).

Computational off-target prediction algorithms are limited and often biased toward nuclease-based editing. Cas-OFFinder was designed to predict nuclease editing, which did not truly reflect base editing with Cas9 nickase activity (65). More recently developed methods such as CHANGE-seq-BE and Selict-seq provide comprehensive, genome-wide analyses of base editor specificity (66, 67). Applying these approaches to patient-derived genomic DNA or iPSC-derived cells will be crucial for accurate off-target risk assessment. Previous studies often used nucleofection of base editors into hiPSCs followed by differentiation into CMs, but editing outcomes may differ across cell types, limiting the physiological relevance of this approach (8, 68). In contrast, our use of mature hiPSC-CMs improves translational applicability. Overall, SlugABE8e and SlugHF-ABE8e(V106W) represent promising, high-specificity candidates for therapeutic development. However, in vivo validation and genome-wide off-target profiling of SlugHF-ABE8e(V106W) will be needed to determine whether it offers potential advantages over SlugABE8e.

In summary, we demonstrated proof-of-concept systemic single-AAV delivery of ABE components in a humanized DMD mouse model. The combination of SlugABE8e, human exon 45 sgRNA, and a myotropic, liver de-targeted MyoAAV capsid offers a powerful and potentially translatable strategy for DMD therapy.

## Methods

### Sex as a biological statement.

Our study exclusively examined cells from male donors and male mice because the disease phenotype modeled primarily affects males.

### Plasmids and vector construction.

The plasmids for SaABE8e (Addgene plasmid 138500), SaKKH-ABE8e (Addgene plasmid 138502), eNme2-C-ABE8e (Addgene plasmid 185667), eNme2-T.1-ABE8e (Addgene plasmid 185668), and SpABE8e (Addgene plasmid 138489) were gifts from David Liu (Harvard University, Cambridge, Massachusetts, USA). The plasmids for Nme2.Smu-ABE8e-i1 (Addgene plasmid 201520), Nme2.Smu-ABE8e-i8 (Addgene plasmid 201522), and Nme2Smu-ABE8e-i1-L10-E932R (Addgene plasmid 228729) were gifts from Erik Sontheimer (University of Massachusetts Chan Medical School, Worcester, Massachusetts, USA). The plasmid for SlugABE-NNG (Addgene plasmid 214356) was a gift from Yongming Wang (Fudan University, Shanghai, China). The plasmid for SlugABE8e-NNGG was constructed from several plasmids: SlugCas9 cDNA from pUC19-E6MBW6 (Addgene plasmid 103102), a gift from Duhee Bang (Yonsei University, Seoul, South Korea); PCR amplification that incorporated the D10A mutation using the primers in Supplemental Table 2; P2A-EGFP from PX458 (Addgene plasmid), a gift from Feng Zhang (Massachusetts Institute of Technology, Cambridge, Massachusetts, USA); and ABE8e from SpABE8e (Addgene plasmid 138489). The sgRNAs for NmeABE were cloned into the NmCas9-sgRNA-expression-in-pBluescript (Addgene plasmid 122091), which was a gift from Scot Wolfe (University of Massachusetts Chan Medical School, Worcester, Massachusetts, USA). The sgRNAs for SaABE8e, SaKKH-ABE8e, SlugABE8e, and eSlugABE8e were cloned into pU6-tevopreq1-GG-acceptor (Addgene plasmid 174038), which was a gift from David Liu. The plasmids for all-in-one AAV constructs were synthesized by Twist Bioscience and cloned into v3em-Nterm-PE2max-digested AAV backbone (Addgene plasmid 198734), a gift from David Liu. Cloning was done using Golden Gate Assembly into restriction enzyme-digested destination vectors. Mutagenesis was performed in the AAV-SlugABE8e plasmid to generate the AAV-SlugHF-ABE8e(V106W) plasmid by HiFi DNA Assembly (NEBuilder) using the primers in Supplemental Table 2.

### In vitro base editing in HEK293T cells.

HEK293T cells (CRL-3216, ATCC) were seeded into 96-well plates at a density of 2.5 × 10^4^ cells per well. 200 ng base editor plasmids and 50 ng sgRNA plasmids were transfected using Lipofectamine 2000 (Thermo Fisher Scientific) according to the manufacturer’s protocol. Cells were harvested 3 days after transfection for Sanger sequencing.

### Genomic DNA and RNA isolation and RT-PCR.

Genomic DNA from hiPSC-CMs was extracted using DirectPCR cell lysis reagent (Viagen) with proteinase K. Genomic DNA of mouse hearts was isolated using a DNeasy Blood & Tissue Kit (Qiagen). RNA from mouse tissues was extracted using TRIzol (Invitrogen). iScript Reverse Transcription Supermix (Bio-Rad) was used to reverse transcribe RNA into cDNA. For RT-PCR, cDNA was used to amplify target exons using GoTaq polymerase (Promega) with primers listed in Supplemental Table 2. Amplicons were run on the gel or sent for Sanger sequencing and 2200 TapeStation analysis.

### Quantitative reverse transcription PCR.

cDNA was used to amplify base editor and sgRNA using KAPA SYBR Fast qPCR Master (Mix KAPA) or TaqMan Fast Advanced Master Mix (Thermo Fisher Scientific) and KiCqStart SYBR Green Primers (Millipore Sigma) listed in Supplemental Table 2 on the Applied Biosystems QuantStudio5 (Thermo Fisher Scientific). Thermal cycling was performed with an initial hold at 50°C for 2 minutes and 95°C for 10 minutes, followed by 40 cycles of 95°C for 15 seconds and 60°C for 1 minute. Gene expression levels were first normalized to the internal control gene *Rplp0* or *Gusb* and then expressed relative to the MyoAAV4E-off group using the 2^–ΔΔCt^ method.

### On-target and off-target editing efficiency analysis.

For Sanger sequencing, DNA was amplified using KAPA2G Fast HotStart Genotyping Mix (Roche) with the primers listed in Supplemental Table 2. Chromatograms were analyzed using EditR to assess gene editing efficiency. For deep amplicon sequencing, candidate off-target sites were identified from Cas-OFFinder, by the number of mismatches, and the top 20 sites were PCR amplified using the primers listed in Supplemental Table 2. Target sites were amplified using PrimeStar GXL polymerase, and a second round of PCR was performed to add Illumina flow cell binding adapters and barcodes. PCR products were purified using AMPure XP beads (Beckman Coulter), analyzed by the 2200 TapeStation system (Agilent) and quantified by the Qubit dsDNA high-sensitivity assay (Invitrogen) before pooling together for Illumina MiSeq. After demultiplexing, resulting reads were analyzed with CRISPResso2 for nucleotide frequency (69).

### Human iPSC maintenance and differentiation.

Healthy iPSCs and DMD ΔEx44 iPSCs were reprogrammed from peripheral blood mononuclear cells from patients at the UT Southwestern Wellstone Myoediting Core as previously described (70). iPSCs were maintained on Growth Factor Reduced Matrigel-coated (Corning) polystyrene tissue culture plates and cultured in mTeSR Plus (STEMCELL). iPSCs were passaged using Versene (Thermo Fisher Scientific) when they reached 60%–80% confluency. Cells at 60%–80% confluency were differentiated into CMs as previously described (71). Briefly, at day 0, cells were treated with CHIR99021 (Selleckchem) in RPMI supplemented with ascorbic acid and B27 without insulin (RPMI/B27 minus). At day 1, the medium was replaced with RPMI/B27 minus medium. At day 3, cells were treated with WNT-C59 (Selleckchem) in RPMI/B27 minus medium. At day 5, the medium was replaced with RPMI/B27 minus. At day 7, the medium was replaced with RPMI supplemented with ascorbic acid and B27 (RPMI/B27). From days 10 to 16, CMs were purified using lactate selection medium (RPMI without glucose, sodium DL-lactate, and CDM3). After metabolic selection, hiPSC-CMs were maintained in RPMI/B27, and medium was changed every 3–4 days until use. hiPSC-CMs were dissociated into single cells using 10X TrypLE (Thermo Fisher Scientific) for seeding. CMs at day >30 were used in all studies.

### AAV production and viral copy number assay.

MyoAAV4A and MyoAAV4E-liver off were prepared by the Gene Transfer Vector Core from Mass General Brigham Hospital. For AAV6 production, AAVpro 293T cells (Clontech Labs) were plated in 15 cm dishes at a density of 1 × 10^7^ cells/dish. The following day, each plate was transfected with 10 μg pHelper, 10 μg pRepCap6 (Addgene plasmid 110770), and 10 μg ITR plasmid using 3 μg PEI per 1 μg plasmid DNA. Recombinant AAV was then purified using chloroform precipitation as described in Negrini et al. (72). Titers were calculated using SYBR Green qPCR with AAV2 ITR primers (Supplemental Table 2). AAV viral copy number was determined by digital PCR of purified genomic DNA using custom-designed primers and probes (Integrated DNA Technologies) (Supplemental Table 2) on a QuantStudio Absolute Q digital PCR system (Thermo Fisher Scientific). A copy number reference assay for *Gapdh* was used to normalize genome copy number.

### In vitro base editing in hiPSC-CMs.

For immunocytochemistry and on-target editing, healthy or DMD ΔEx44 iPSC-CMs were seeded into 24-well plates at a density of 1 × 10^5^ cells per well. 600 ng of base editor plasmids and 200 ng of sgRNA plasmids were transfected into CMs using ViaFect (Promega, E4981). Cells were harvested 5 days after transfection for Sanger sequencing. For all-in-one AAV6 transduction, DMD ΔEx44 hiPSC-CMs were treated at a dose of 1 × 10^5^ vg/cell for 4 days and were harvested after 20–30 days of transduction for downstream analysis.

### Western blot.

DMD hiPSC-CMs were lysed in lysis buffer (10% SDS, 62.5 mM Tris [pH 6.8], 1 mM EDTA [pH 8.0]). Tissues were crushed into fine powder using a liquid nitrogen–frozen crushing apparatus and then lysed in lysis buffer. Muscle lysates were transferred into lysing tubes containing 1.4-mm ceramic beads (Lysing Matrix D, MP Biomedicals) for homogenizing. Protein concentration was measured by a BCA Protein Assay Kit (Thermo Fisher Scientific). 15 μg of DMD hiPSC-CMs protein or 50 μg of DMD mouse protein was loaded onto a 4%–20% Criterion TGX Precast Midi Protein Gel (Bio-Rad). The gel was run at 80 V for 30 minutes, 100 V for another 30 minutes, and 120 V for 2 hours, followed by transfer to a PVDF membrane at 100 V at 4°C for 1.5 hours. The PVDF membrane was blocked in blocking buffer (5% nonfat dry milk in Tris buffered saline with Tween 20), and was then incubated with mouse anti-dystrophin (1:1,000) (D8168/MANDYS8, Sigma-Aldrich) overnight at 4^o^C or mouse anti-vinculin (1:1,000) (V9131/hVIN-1, Sigma-Aldrich) for 1 hour at room temperature, followed by goat anti-mouse IgG (H+L)-HRP conjugate (1:10,000) (1706516, Bio-Rad) for 1 hour at room temperature. The PVDF membrane was developed by Western Blotting Luminol Reagent (sc2048, Santa Cruz Biotechnology). Images were captured by the ChemiDoc MP Imaging System (Bio-Rad). Relative protein expression was quantified using ImageJ (NIH).

### Immunofluorescence staining.

hiPSC-CMs were grown on 12 mm coverslips coated with poly-D-lysine and Growth Factor–Reduced Matrigel (Corning). After AAV6 treatment, cells were fixed with cold acetone for 15 minutes at –20°C and permeabilized with 0.3% Triton X-100 (Thermo Fisher Scientific) for 15 minutes at room temperature. Coverslips were blocked with 5% goat serum/PBS for 1 hour. Cells were incubated with mouse anti-dystrophin (1:800) (D8168/MANDYS8, Sigma-Aldrich) and rabbit anti-troponin I (1:500) (sc15368, Santa Cruz Biotechnology) antibodies in 5% goat serum/PBS overnight at 4°C. The next day, cells were incubated with goat anti-mouse IgG (H+L) Alexa Fluor 555 (A-21424, Invitrogen), goat anti-rabbit IgG (H+L) Alexa Fluor 488 (A-11008, Invitrogen), and DAPI (D9542, Sigma-Aldrich) for 1 hour. For tissues, transverse 10 μm cryosections of skeletal muscle and cardiac tissue were prepared. Sections were rinsed twice with PBS at room temperature, followed by a 10-minute 4% PFA fixation and a 30-minute delipidation step in 0.3% Triton X-100 in PBS. Slides were then washed again, twice, with PBS and once with 0.01% Triton X-100 in PBS. After washing, sections were washed and blocked for 1 hour each with mouse IgG blocking reagent and 8% M.O.M protein concentrate (M.O.M. Kit, Vector Laboratories) in 5% goat serum/PBS (MilliporeSigma) and then incubated overnight at 4°C with a mouse anti-dystrophin antibody (D8168/MANDYS8, Sigma- Aldrich). Following primary antibody incubation, sections were washed, twice, with PBS and once with 0.01% Triton X-100 in PBS, and incubated with goat anti-mouse IgG (H+L) Alexa Fluor 488 (A-11001, Invitrogen), goat anti-rabbit IgG (H+L) Alexa Fluor 555 (A-21428, Invitrogen), and DAPI for 1 hour. Coverslips and sections were mounted on Superfrost Plus microscope slides (Fisherbrand) with Epredia Immu-Mount (Fisher Scientific). Confocal images were captured using a Zeiss LSM 800 microscope.

### Mice.

C57BL/6N WT male mice (strain 556) were ordered from Charles River. The Δ44; h45 DMD mouse model was generated in the C57BL/6N background and was previously published (37). For retro-orbital injections, mice were first anesthetized by intraperitoneal injection of 2.5% 2,2,2-Tribromoethanol (Sigma-Aldrich). 5 × 10^13^ vg/kg all-in-one AAVs in saline solution were injected into 4-week-old Δ44;h45 mice through the retro-orbital sinus using BD Ultra-Fine Insulin Syringes 31G. Tissues and serum were collected after 4 weeks of AAV treatment and functional testing for downstream analysis.

### Grip-strength tests, treadmill exercise test, and serum protein measurements.

Forelimb-muscle strength of WT, saline-treated Δ44;h45, and AAV-treated Δ44;h45 mice was assessed by the grip-strength meter (Columbus Instruments). Briefly, mice were weighed and lifted by the tail to grasp the metal grip that connected to the grip-strength meter. The peak force in grams was recorded. Grip-strength tests were repeated 5 times for individual mice at 10-second intervals. The treadmill exercise was conducted using an Exer-6M (Columbus Instruments) at 15 degrees downhill. Before the test, mice were placed on the treadmill and allowed to warm up for 5 minutes at 5 m/min. For the exercise, mice ran on the treadmill for 2 minutes at 5 m/min, 2 minutes at 7 m/min, 2 minutes at 8 m/min, and 5 minutes at 10 m/min. Afterward, the speed was increased by 1 m/min until a maximum speed of 20 m/min was reached. Mice were considered exhausted when they were unable to maintain running on the treadmill despite repeated electrical prodding. Sera were collected, and CK, ALT, AST, ALKP, and TBIL were measured by the Metabolic Phenotyping Core at UT Southwestern Medical Center using the VITROS 350 Chemistry System.

### Tissue histology and quantification.

Hearts were embedded in tissue freezing medium (General Data Healthcare) and snap-frozen in dry ice. Skeletal muscles were cryo-embedded in a 1:2 (vol:vol) mixture of tragacanth (Sigma-Aldrich) to tissue freezing medium and snap-frozen in isopentane heat extractant and supercooled to –155°C. Tissues were cryosectioned into 10 μm sections. H&E, Picrosirius red, and Von Kossa staining were performed according to established protocols. Images were acquired from a Keyence BZ-X700 microscope. H&E images were used to quantify centralized nuclei. PSR images were used to quantify collagen areas. Laminin immunostaining images were used to quantify myofiber cross-sectional area distribution. Quantification was done using ImageJ (NIH).

### Statistics.

Data analyses were performed with GraphPad Prism 10.0.1. Unpaired 2-tailed Student’s *t* tests were performed for comparison between the two groups. One-way ANOVA with Tukey’s multiple comparisons test was performed to analyze 3 or more groups. All data are presented as mean ± SD, with the exception of Figure 6B, which is presented as mean ± SEM. *P* values of less than 0.05 were considered statistically significant.

### Study approval.

All animal experiments were approved under protocols (2015-100829, 2016-101833, and 2017-101971) by the Institutional Animal Care and Use Committee at the University of Texas Southwestern Medical Center. All animals were housed in a 12-hour-light/dark cycle with a temperature of 22°C and supplied with standard chow in a pathogen-free facility.

### Data availability.

All data presented in this study are available in the Supporting Data Values file.

## Manuscript information

### Author contributions.

YCP, NL, and ENO designed research. YCP, H Li, GB, MZD, ZX, ACC, FC, and ESO performed research. H Liu, NZ, and LHV contributed new reagents. YCP, H Li, and GB analyzed data. YCP, NL, and ENO wrote the paper. All authors discussed the results and participated in the preparation and editing of the article. The order of first authors was decided by internal discussion.

### AI disclosure.

The authors declare that no AI tools were used in the original research for or writing of this article.

### Conflict of interest.

ENO is a consultant for Vertex Pharmaceuticals, Prime Medicine, Cardurion Pharmaceuticals, and Tenaya Therapeutics. LHV is a paid advisor for Novartis, Akouos, and Affinia Therapeutics and serves on the board of directors of Affinia, Addgene, and Odylia Therapeutics. LHV holds equity in Akouos and Affinia and receives sponsored research funding from Albamunity, to which he is an unpaid consultant. LHV is a scientific advisory board member of Akouos and board member of Affinia Therapeutics, companies of which he is a cofounder. NZ receives sponsored research funding from Akouos.

### Funding.

This study was supported by the following:

- NIH grants (R01AR080676 to ENO and NL).
- Senator Paul D. Wellstone Muscular Dystrophy Specialized Research Center (P50HD087351; to ENO).
- Foundation Leducq Transatlantic Networks of Excellence in Cardiovascular Research (to ENO).
- Robert A. Welch Foundation (1-0025; to ENO).
- CureHeart, the British Heart Foundation’s Big Beat Challenge Award (BBC/F/21/220106; to ENO).

This work is the result of NIH funding, in whole or in part, and is subject to the NIH Public Access Policy. Through acceptance of this federal funding, the NIH has been given a right to make the work publicly available in PubMed Central.

## Supplementary Material

Supplemental data

Supporting data values

## Figures and Tables

**Figure 1:**
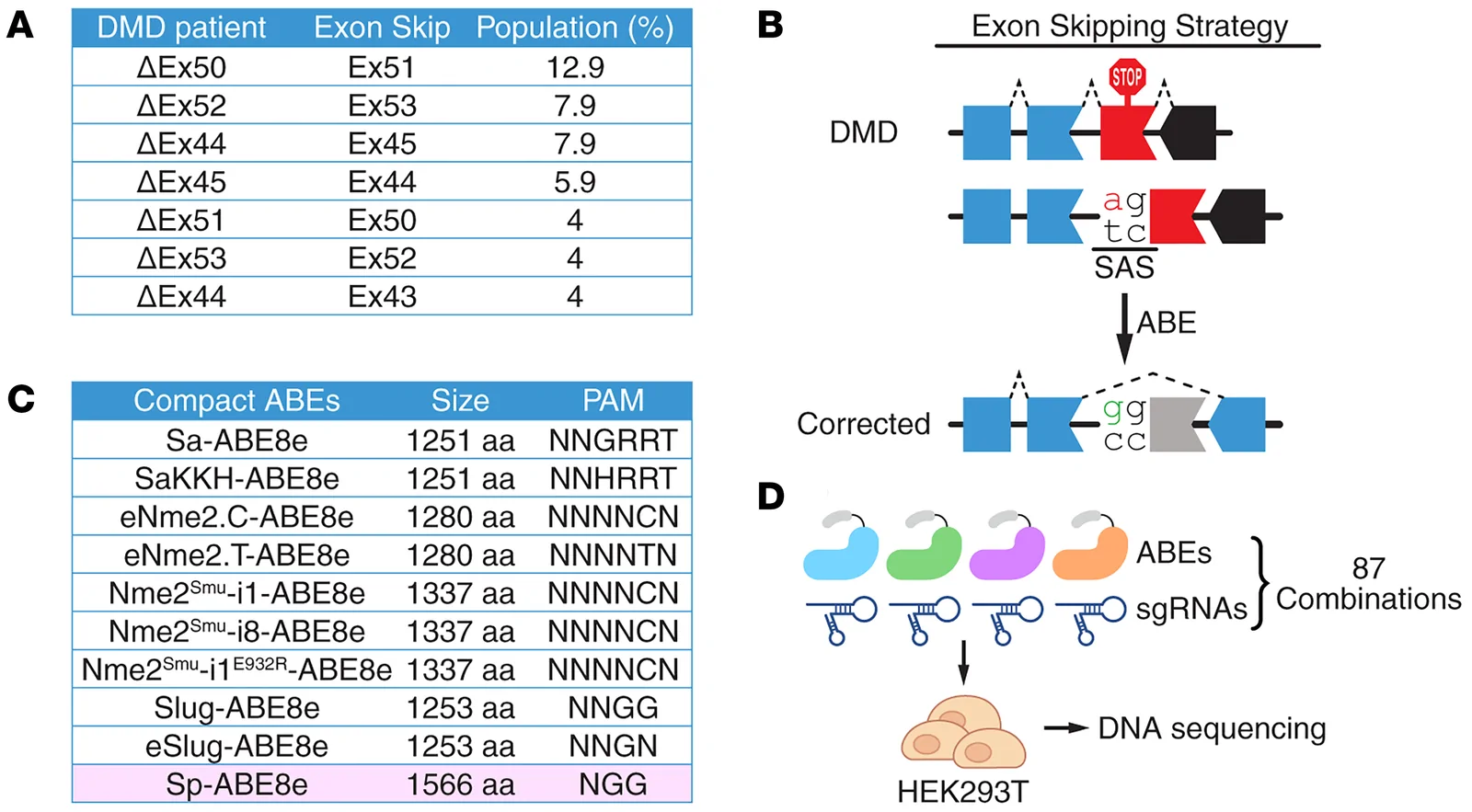
Compact ABE screening for *DMD* exon skipping in HEK293T cells. (**A**) Proportions of patients with DMD who are amenable to the base-editing exon-skipping strategy. Theoretical *DMD* deletions in patients for exon skipping by CureDuchenne (73) were used. (**B**) Adenine base editing (ABE) performs a single-swap “ag”-to-“gg” nucleotide change to the splicing acceptor site (SAS) to skip out-of-frame exons or exons with a premature stop codon and reframe the *DMD* open reading frame. (**C**) Comparison of protein size and PAM sequences of compact ABE8e systems with Sp-ABE8e. (**D**) Screening of 87 compact ABE and sgRNA combinations in HEK293T cells. Cells were harvested 3 days after ABE transfection, and editing efficiency was determined by Sanger sequencing.

**Figure 2:**
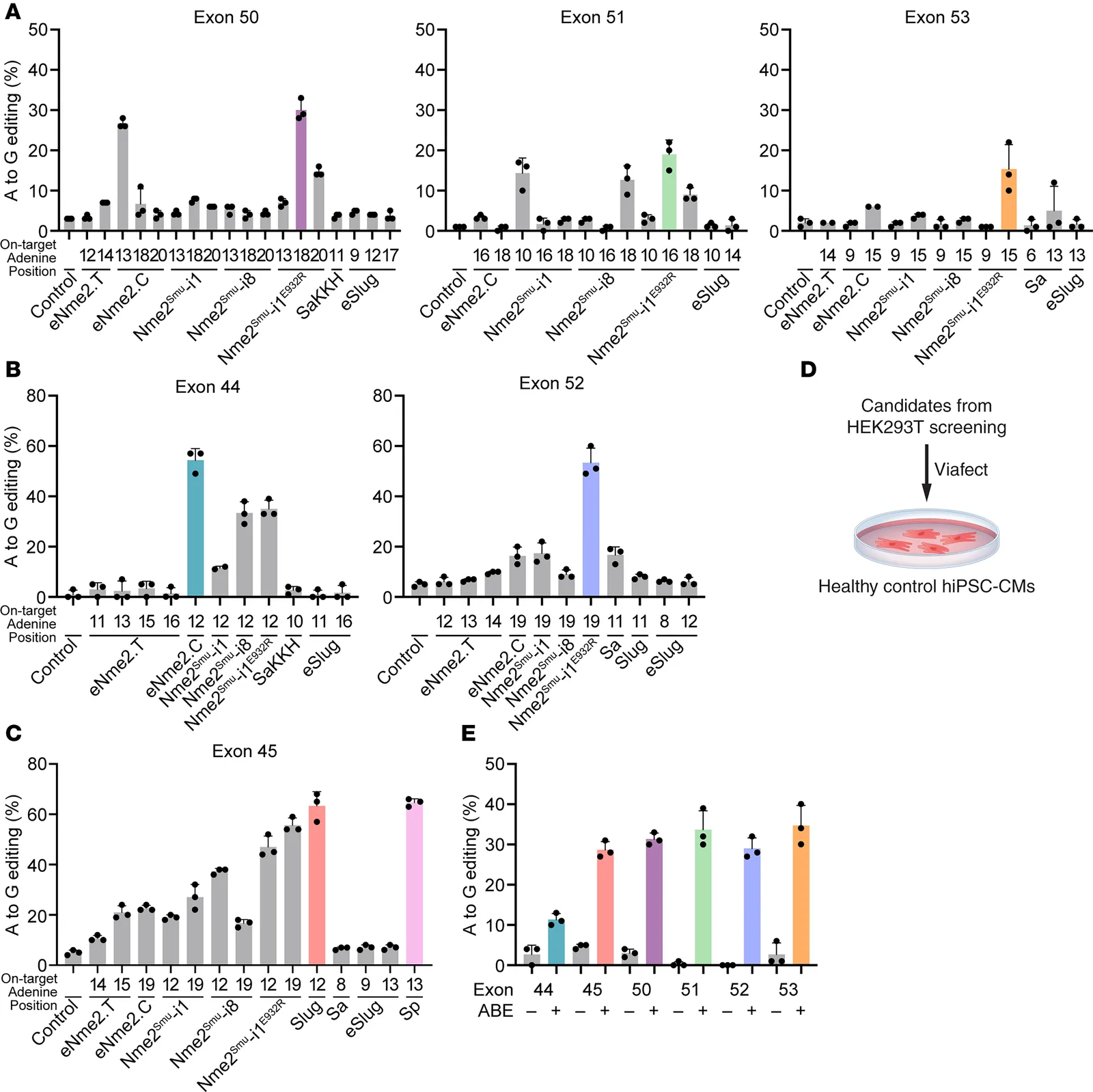
Mini-ABE–mediated A-to-G editing in the SAS of hotspot *DMD* exons in HEK293T cells and hiPSC-CMs. Percentage of on-target A-to-G DNA editing in SAS of *DMD* exon 50, 51, and 53 (**A**), exon 44 and exon 52 (**B**), and exon 45 (**C**) in HEK293T cells was calculated based on Sanger sequencing results. For **A**–**C**, ABEs and their sgRNAs with on-target adenine position are shown on the *x* axis. Control samples were transfected with Lipofectamine 2000 only. A published Sp-ABE8e and sgRNA pair (pink) was used as a comparison in exon 45 (8). (**D**) Top combinations from the HEK293T cell screening were transfected into healthy control hiPSC-CMs by Viafect, and cells were cultured for 5 days. (**E**) Percentage of SAS A-to-G DNA editing in *DMD* exon 44, 45, 50, 51, 52, and 53 by Sanger sequencing in hiPSC-CMs (*n* = 2–3 biologically independent replicates). “–” denotes the use of Viafect only, and “+” denotes the use of a base editor and a sgRNA. Data are presented as mean ± SD.

**Figure 3:**
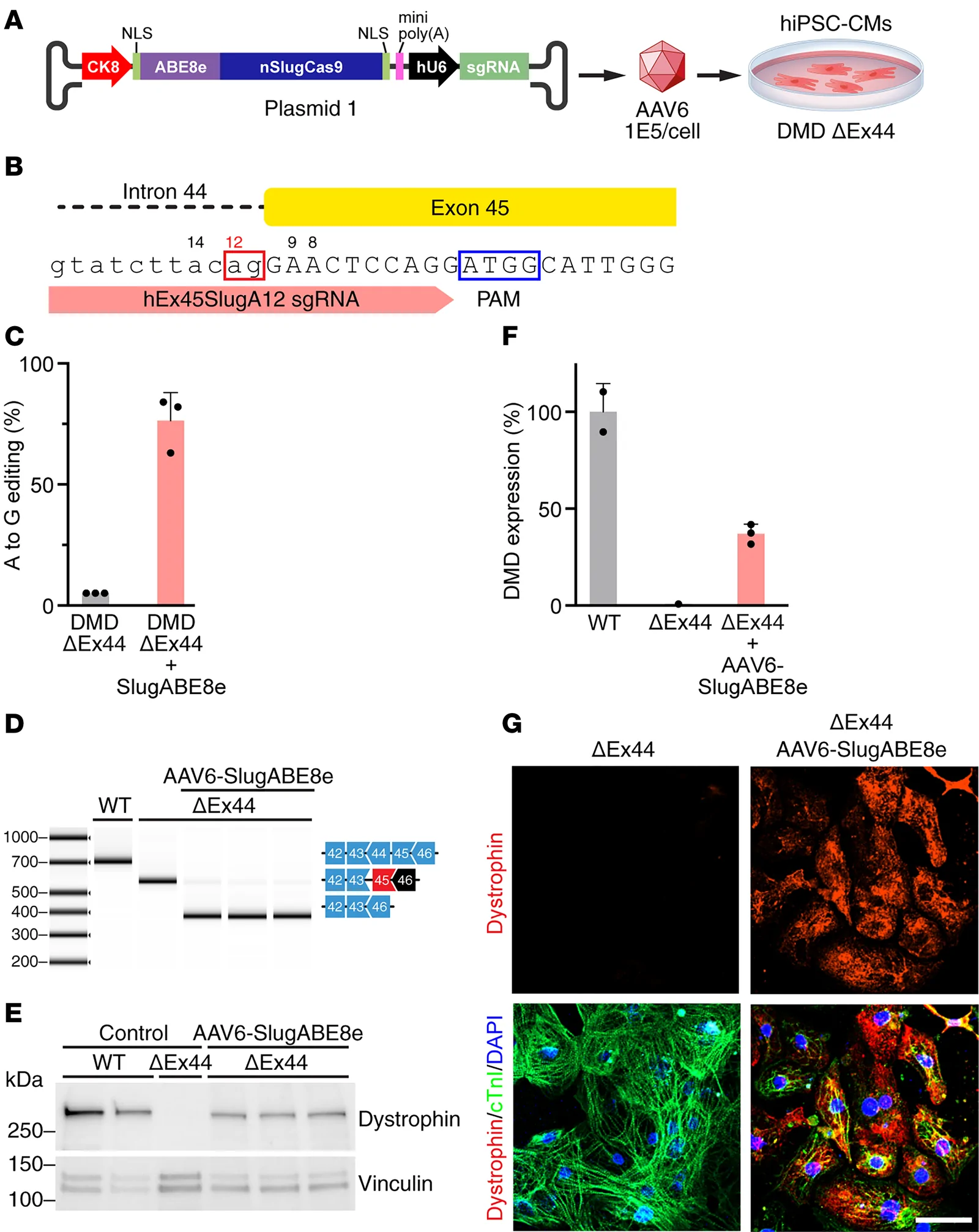
All-in-one AAV6-SlugABE8e induced exon skipping and restoration of dystrophin expression in DMD ΔEx44 hiPSC-CMs. (**A**) Schematic of Slug-ABE8e AAV construct. A muscle-specific CK8e promoter and a human U6 promoter were used to drive Slug-ABE8e and sgRNA expression in the AAV vector, respectively. Differentiated ΔEx44 hiPSC-CMs were treated with 1 × 10^5^ vg/cell of AAV6-SlugABE8e for 3 weeks. (**B**) Schematic of hEx45SlugA12 sgRNA for Slug-ABE8e exon 45 skipping. Target adenine (A12) is shown in the red box, and bystander adenines (A8, A9, and A14) are labeled. The PAM sequence is shown in the blue box. (**C**) On-target DNA editing of AAV6-SlugABE8e–treated DMD ΔEx44 hiPSC-CMs (*n* = 3) detected by Sanger sequencing. (**D**) RT-PCR analysis of AAV6-SlugABE8e–treated DMD ΔEx44 hiPSC-CMs. Corresponding exon structures are indicated on the right. (**E**) Western blot showing that AAV6-SlugABE8e–treated DMD ΔEx44 hiPSC-CMs partially restored dystrophin expression. Vinculin was the loading control. (**F**) Quantification of dystrophin expression in isogenic control, ΔEx44, and AAV6-SlugABE8e–treated ΔEx44 hiPSC-CMs. Relative intensity was measured as dystrophin expression normalized to vinculin expression compared with the isogenic control hiPSC-CMs. (**G**) Immunocytochemistry of AAV6-SlugABE8e–treated ΔEx44 hiPSC-CMs (*n* = 3). Dystrophin is shown in red; cardiac troponin I (cTnI) highlights CMs in green; DAPI stains for nuclei in blue. Scale bar: 50 μm. Data are presented as mean ± SD. For RT-PCR (**D**), WT (*n* = 1), ΔEx44 (*n* = 1), and ΔEx44+AAV6 (*n* = 3). For Western blot (**E** and **F**), WT (*n* = 2), ΔEx44 (*n* = 1), and ΔEx44+AAV6 (*n* = 3).

**Figure 4:**
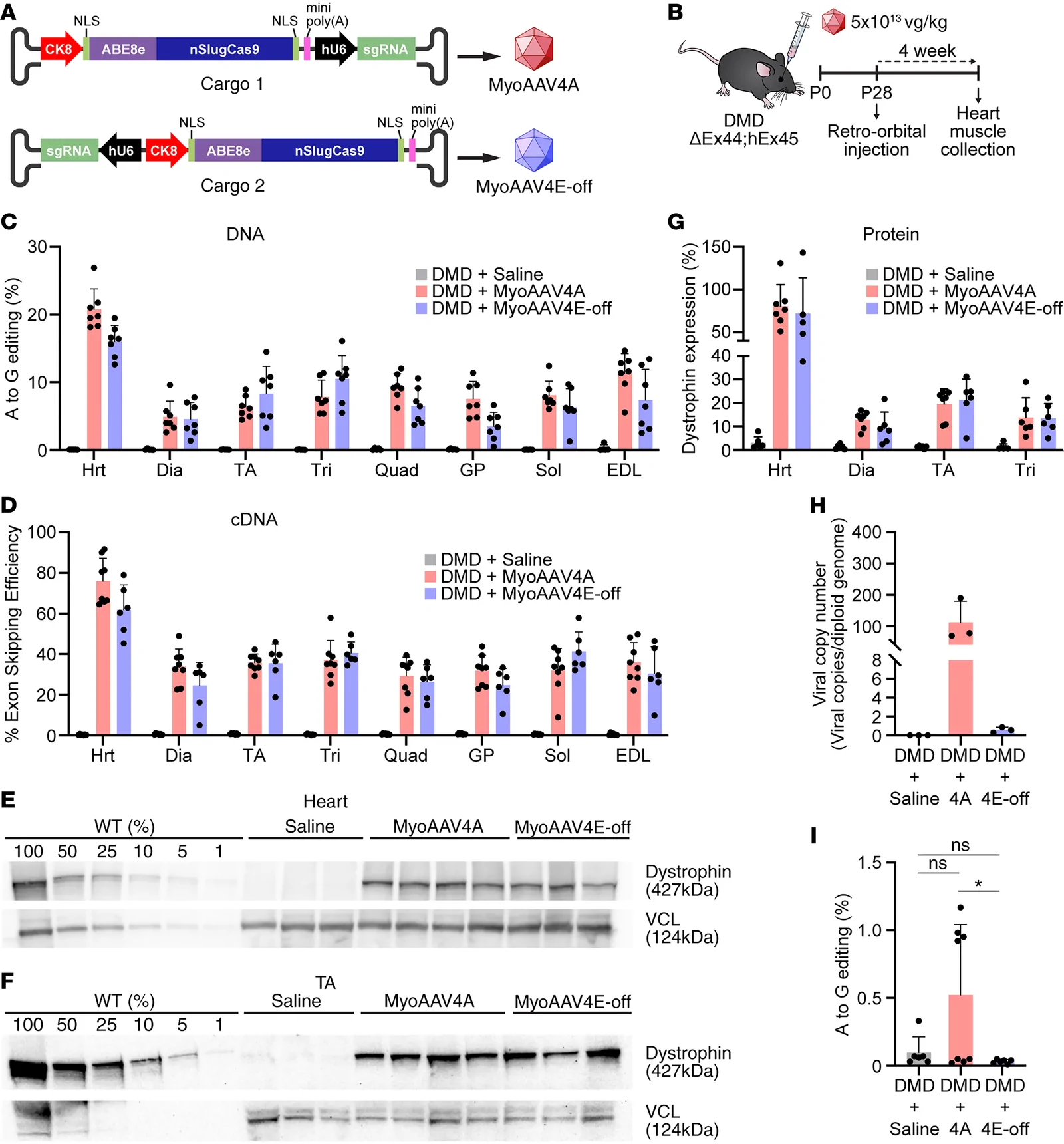
MyoAAV4-mediated exon skipping in juvenile ΔEx44;hEx45 DMD mice. (**A**) Schematic of Slug-ABE8e AAV constructs. Cargo 1 contained CK8e-SlugABE8e and hU6-sgRNA cassettes in a tandem orientation, while these cassettes were in an inverted orientation in Cargo 2. Cargo 1 was packaged into MyoAAV4A, and cargo 2 was packaged into MyoAAV4E- off. (**B**) At P28, ΔEx44;hEx45 DMD mice were administered with saline, MyoAAV4A, or MyoAAV4E-off through retro-orbital injection. Four weeks after injection, tissues were harvested. (**C**) DNA editing efficiency as determined by deep amplicon sequencing of genomic DNA from ΔEx44;hEx45 heart (Hrt), diaphragm (Dia), tibialis anterior (TA), triceps (Tri), quadriceps (Quad), gastrocnemius-plantaris (GP), soleus (Sol), and extensor digitorum longus (EDL) with saline (*n* = 5), MyoAAV4A (*n* = 8), or MyoAAV4E-off treatment (*n* = 6). (**D**) Editing efficiency at the cDNA level as determined by deep amplicon sequencing of cDNA in ΔEx44;hEx45 Hrt, Dia, TA, Tri, Quad, GP, Sol, and EDL with saline (*n* = 7), MyoAAV4A (*n* = 8), or MyoAAV4E-off (*n* = 6) treatment. (**E** and **F**) Western blot showing restoration of dystrophin expression in the Hrt and TA. Vinculin (VCL) is the loading control. (**G**) Quantification of dystrophin expression based on Western blot in ΔEx44;hEx45 Hrt, Dia, TA, and Tri with saline (*n* = 6), MyoAAV4A (*n* = 6–7), or MyoAAV4E-off (*n* = 5–6) treatment. Relative intensity was measured as dystrophin expression normalized to vinculin expression compared with WT mice as shown in **E** and **F**. (**H**) Viral copy number of AAV from the liver with saline (*n* = 3), MyoAAV4A (*n* = 3), or MyoAAV4E-off (*n* = 3), normalized to mGapdh by digital PCR. (**I**) DNA editing efficiency as determined by deep amplicon sequencing of genomic DNA in the liver of ΔEx44;hEx45 mice treated with saline (*n* = 5), MyoAAV4A (*n* = 8), or MyoAAV4E-off (*n* = 6) treatment. Data are presented as mean ± SD. *P < 0.05 by ordinary 1-way ANOVA. 4A, MyoAAV4A; 4E-off, MyoAAV4E-off.

**Figure 5:**
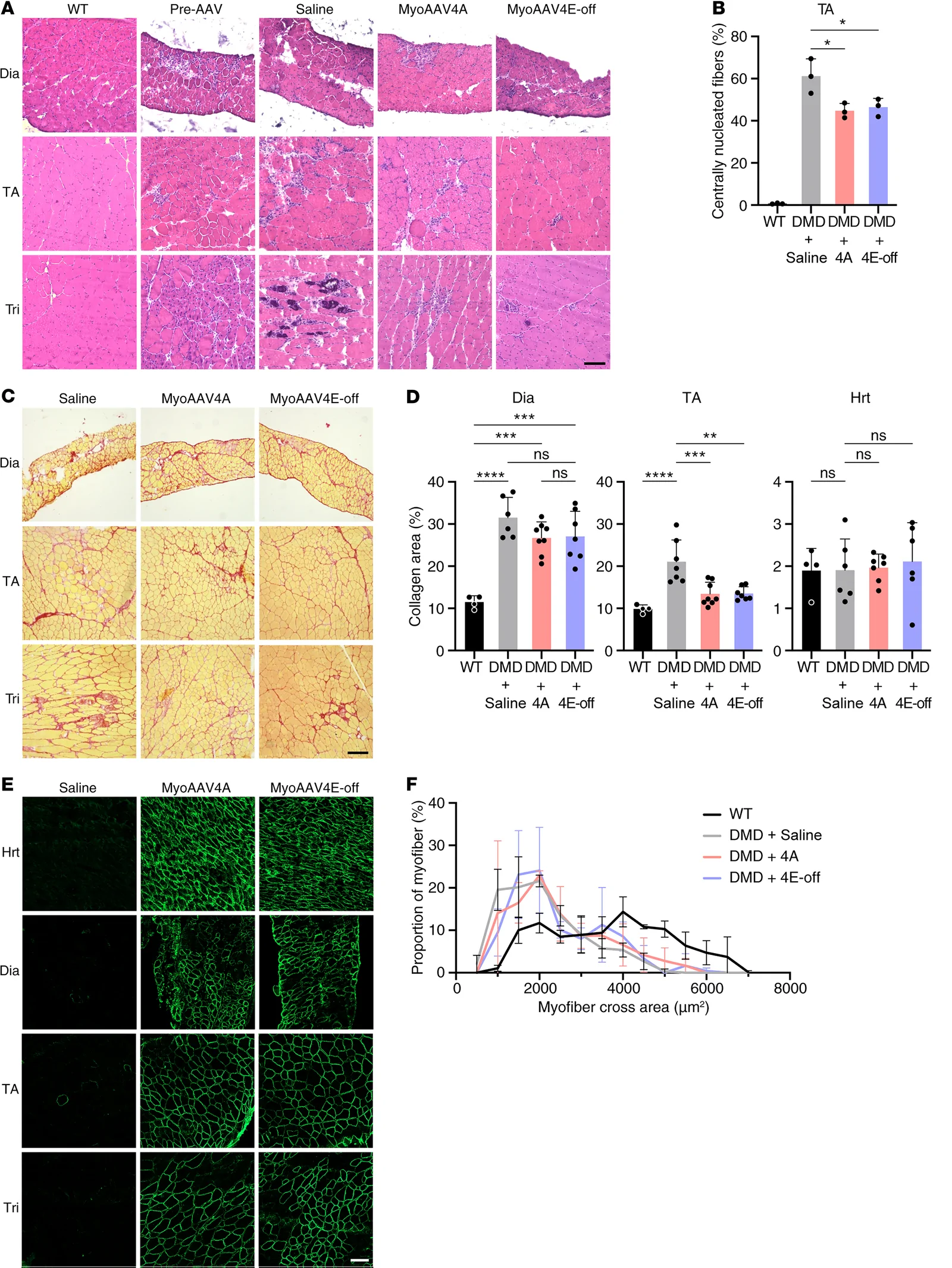
All-in-one Slug-ABE8e delivered by MyoAAV4 enhanced muscle integrity and prevented disease progression in ΔEx44;hEx45 DMD mice. (**A**) H&E staining of Dia, TA, and Tri sections from 4-week-old untreated ΔEx44;hEx45 mice, 8-week-old WT mice and 8-week-old ΔEx44;hEx45 mice treated with saline, MyoAAV4A, or MyoAAV4E-off for 4 weeks. Scale bar: 200 μm. (**B**) Quantification of centrally nucleated myofibers of TA sections from 8-week-old WT mice (*n* = 3) and ΔEx44;hEx45 mice treated with saline (*n* = 3), MyoAAV4A (*n* = 3), or MyoAAV4E-off (*n* = 3). (**C**) Picrosirius red staining of Dia, TA, and Tri sections from 8-week-old WT mice and saline-, MyoAAV4A-, or MyoAAV4E-off–treated ΔEx44;hEx45 mice. Scale bar: 200 μm. (**D**) Quantification of fibrosis of Dia, TA, and Hrt sections from 8-week-old WT mice (*n* = 4) and ΔEx44;hEx45 mice treated with saline (*n* = 6–7), MyoAAV4A (*n* = 8-9), or MyoAAV4E-off (*n* = 7). (**E**) Immunocytochemistry of Hrt, Dia, TA, and Tri sections from WT mice and saline-, MyoAAV4A-, or MyoAAV4E-off–treated ΔEx44;hEx45 mice. Scale bar: 50 μm. Dystrophin is in green. (**F**) Quantification of myofiber cross-sectional area of TA sections from 8-week-old WT mice (*n* = 4) and ΔEx44;hEx45 mice treated with saline (*n* = 3), MyoAAV4A (*n* = 3), or MyoAAV4E-off (*n* = 3). Data are presented as mean ± SD. *P < 0.05, **P < 0.01, ***P < 0.001, ****P < 0.0001 by ordinary 1-way ANOVA. 4A, MyoAAV4A; 4E-off, MyoAAV4E-off.

**Figure 6:**
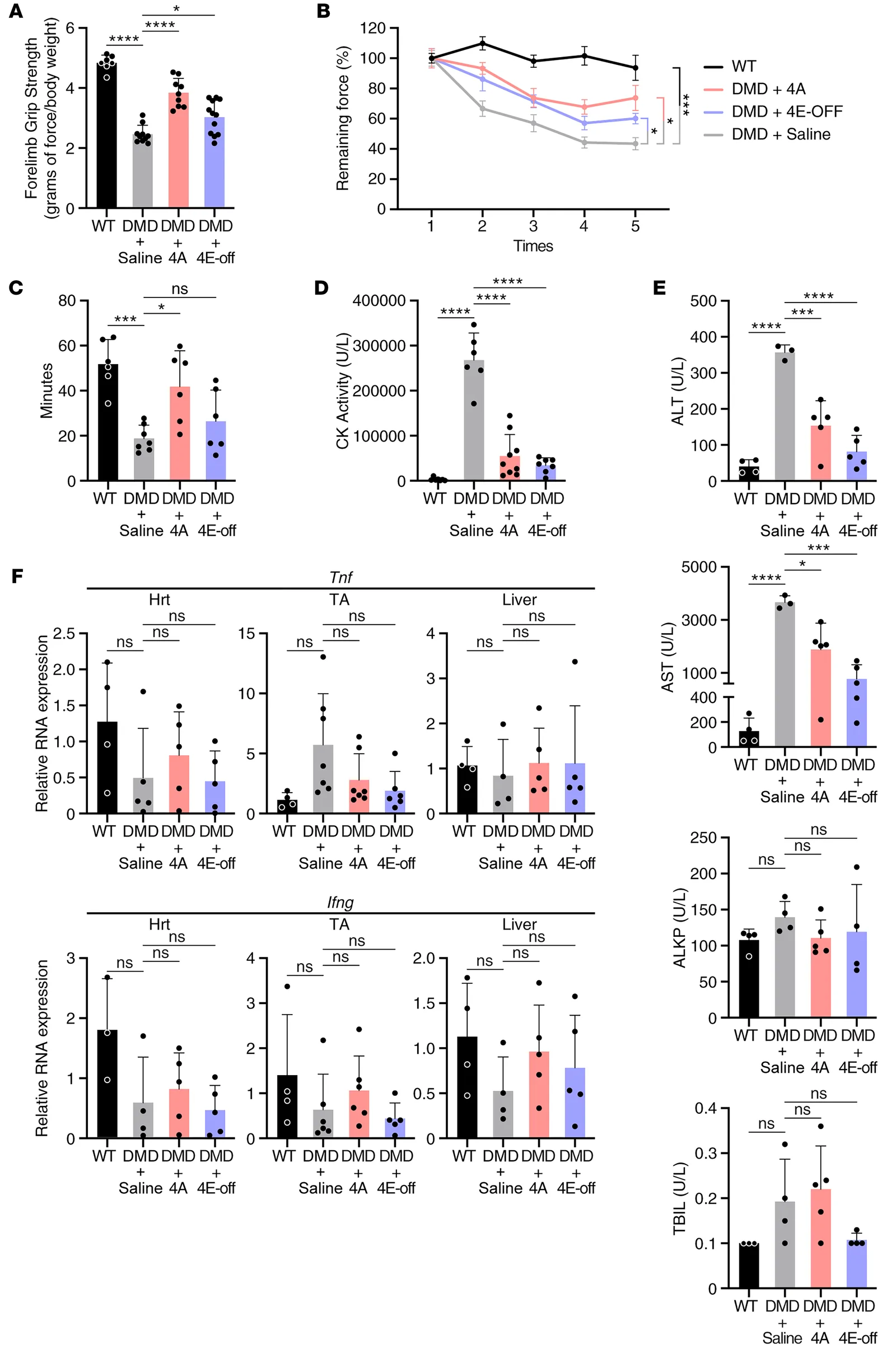
All-in-one Slug-ABE8e delivered by MyoAAV4 enhanced muscle strength and endurance without induced inflammation in ΔEx44;hEx45 DMD mice. (**A**) Forelimb grip strength of 8-week-old WT mice (*n* = 7) and saline- (*n* = 10), MyoAAV4A- (*n* = 9), or MyoAAV4E-off–treated (*n* = 12) ΔEx44;hEx45 mice. Grip strength was normalized to body weight (grams of force per gram of body weight). (**B**) The remaining force was measured with 5 repetitive tests at 10-second intervals. WT mice (*n* = 7), saline (*n* = 10), MyoAAV4A (*n* = 9), and MyoAAV4E-off (*n* = 12) mice. (**C**) WT mice (*n* = 5) and saline- (*n* = 7), MyoAAV4A- (*n* = 6), or MyoAAV4E-off–treated (*n* = 6) ΔEx44;hEx45 mice were subjected to treadmill exercise. Muscle performance was measured as time to exhaustion. (**D**) Serum CK activity in 8-week-old WT (*n* = 7) and saline- (*n* = 6), MyoAAV4A- (*n* = 9), or MyoAAV4E-off–treated (*n* = 7) ΔEx44;hEx45 mice. (**E**) Serum ALT, AST, ALKP, and TBIL in 8-week-old WT (*n* = 3–4) and saline- (*n* = 3–4), MyoAAV4A- (*n* = 5), or MyoAAV4E-off–treated (*n* = 4–5) ΔEx44;hEx45 mice. (**F**) qPCR analysis of *Tnf* and *Ifng* of Hrt, TA, and liver in 8-week-old WT (*n* = 3–4) and saline- (*n* = 4–7), MyoAAV4A- (*n* = 5–7), or MyoAAV4E-off–treated (*n* = 5–6) ΔEx44;hEx45 mice. Data are presented as mean ± SD in **A** and **C**–**F**; data are presented as mean ± SEM in **B**. *P < 0.05, ***P < 0.001, ****P < 0.0001 by ordinary 1-way ANOVA. 4A, MyoAAV4A; 4E-off, MyoAAV4E-off.

**Figure 7:**
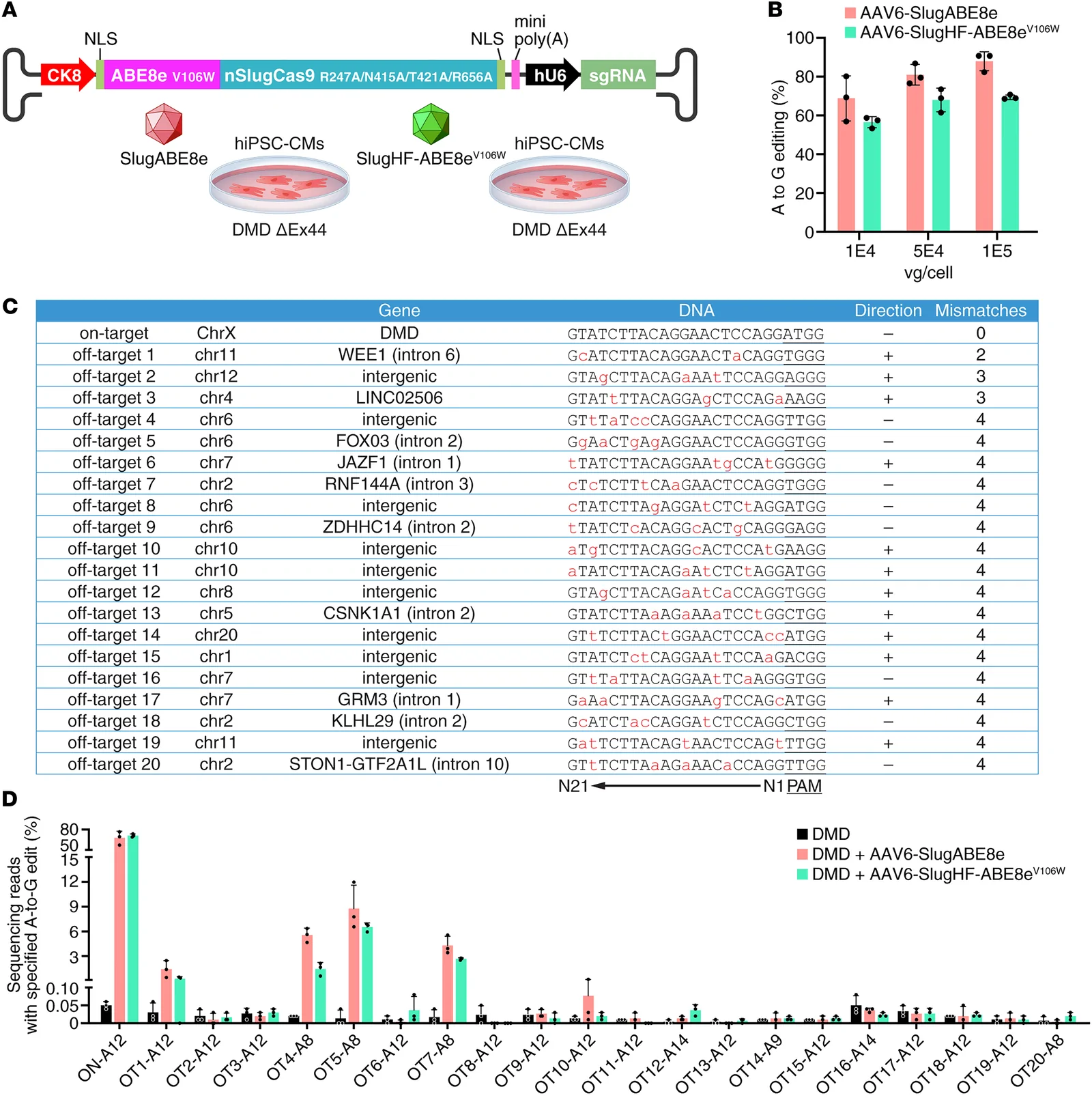
Off-target analysis in ΔEx44 hiPSC-CMs using SlugABE8e and high-fidelity SlugABE variant. (**A**) Schematic of the SlugHF-ABE8e(V106W) AAV construct. Differentiated ΔEx44 hiPSC-CMs were treated with AAV6-SlugABE8e or AAV6-SlugHF-ABE8e(V106W) each with 1 × 10^4^, 5 × 10^4^, and 1 × 10^5^ vg/cell for 3 weeks. (**B**) On-target DNA editing of AAV6-SlugABE8e (*n* = 3) or AAV6-SlugHF-ABE8e(V106W) (*n* = 3) treated DMD ΔEx44 hiPSC-CMs as determined by deep amplicon sequencing. (**C**) Cas-OFFinder prediction of potential off-target sites. Twenty off-target candidates were selected. Mismatches in sgRNA sequences are shown in red. (**D**) Deep amplicon sequencing of Cas-OFFinder off-target sites after AAV6-SlugABE8e (*n* = 3) or AAV6-SlugHF-ABE8e(V106W) (*n* = 3) treatment. Data are presented as mean ± SD.

## Version 1 11/02/2026

Electronic publication
