# Supplementary material for: A single liver-sparing myotropic AAV ameliorates muscular dystrophy in humanized mice by base editing
Source: J Clin Invest. 2026 Nov 2;136(21):e202378. doi: 10.1172/JCI202378 (PMC13722381; doi:10.1172/JCI202378)
Supplement: Supplementary file 1 [file jci-136-202378-s160.pdf]

Supplemental material

A Single Liver-Sparing Myotropic AAV Ameliorates Muscular Dystrophy in Humanized Mice by Base Editing

Yu-Chung Pien[1,2,3†], Hui Li[1,2,3†], Gabby Bulliard[1,2,3], Huachun Liu[4,5], Mateusz Durbacz[1,2,3], Zhisheng Xu[1,2,3], Andreas C. Chai[1,2,3], Francesco Chemello[1,2,3], Efrain Sanchez-Ortiz[1,2,3], Nerea Zabaleta[4,5,6], Luk H. Vandenberghe[4,5], Ning Liu[1,2,3], Eric N. Olson[1,2,3*]

[1]Department of Molecular Biology, Hamon Center for Regenerative Science and Medicine, The University of Texas Southwestern Medical Center, Dallas, Texas, 75390, USA

[2]Hamon Center for Regenerative Science and Medicine, University of Texas Southwestern Medical Center, Dallas, TX 73590, USA

[3]Senator Paul D. Wellstone Muscular Dystrophy Specialized Research Center, University of Texas Southwestern Medical Center, Dallas, TX 75390, USA

[4]Grousbeck Gene Therapy Center, Schepens Eye Research Institute, and Massachusetts Eye and Ear Infirmary, Harvard Medical School, Boston, MA, 02114, USA

[5]The Broad Institute of Harvard and MIT, Cambridge, MA, 02142, USA

[6]Cima Universidad de Navarra, Pamplona, 31014, Spain

†These authors share the first authorship.

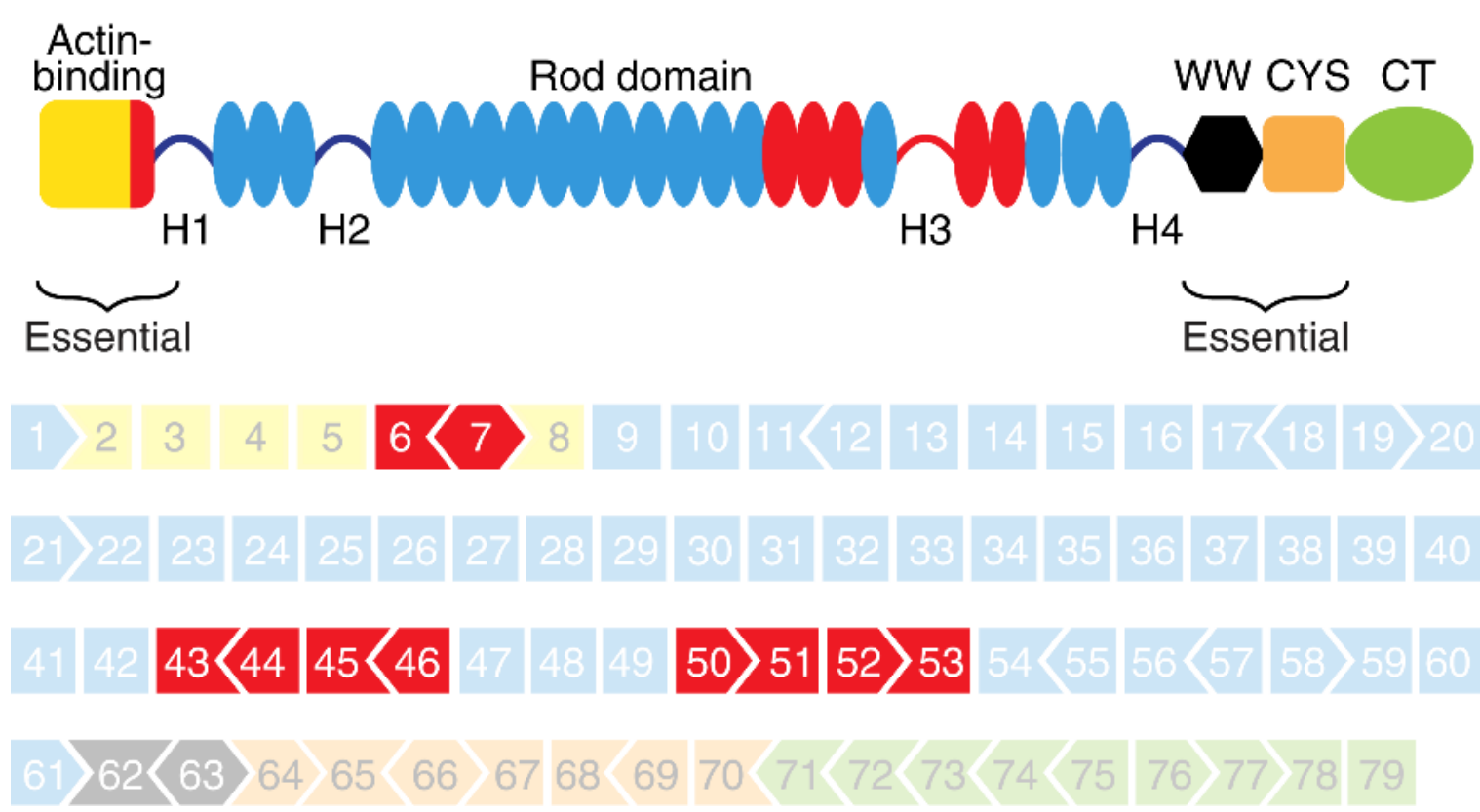

Supplemental Figure 1. Mutational hotspots in the *DMD* gene. Top: dystrophin protein domains. Color coded regions reflect encoding exons. Bottom: exons 6-7, exons 43-46, and exons 50-53 have been identified as mutational hotspots. Hotspots in the protein and gene structure are shown in red.

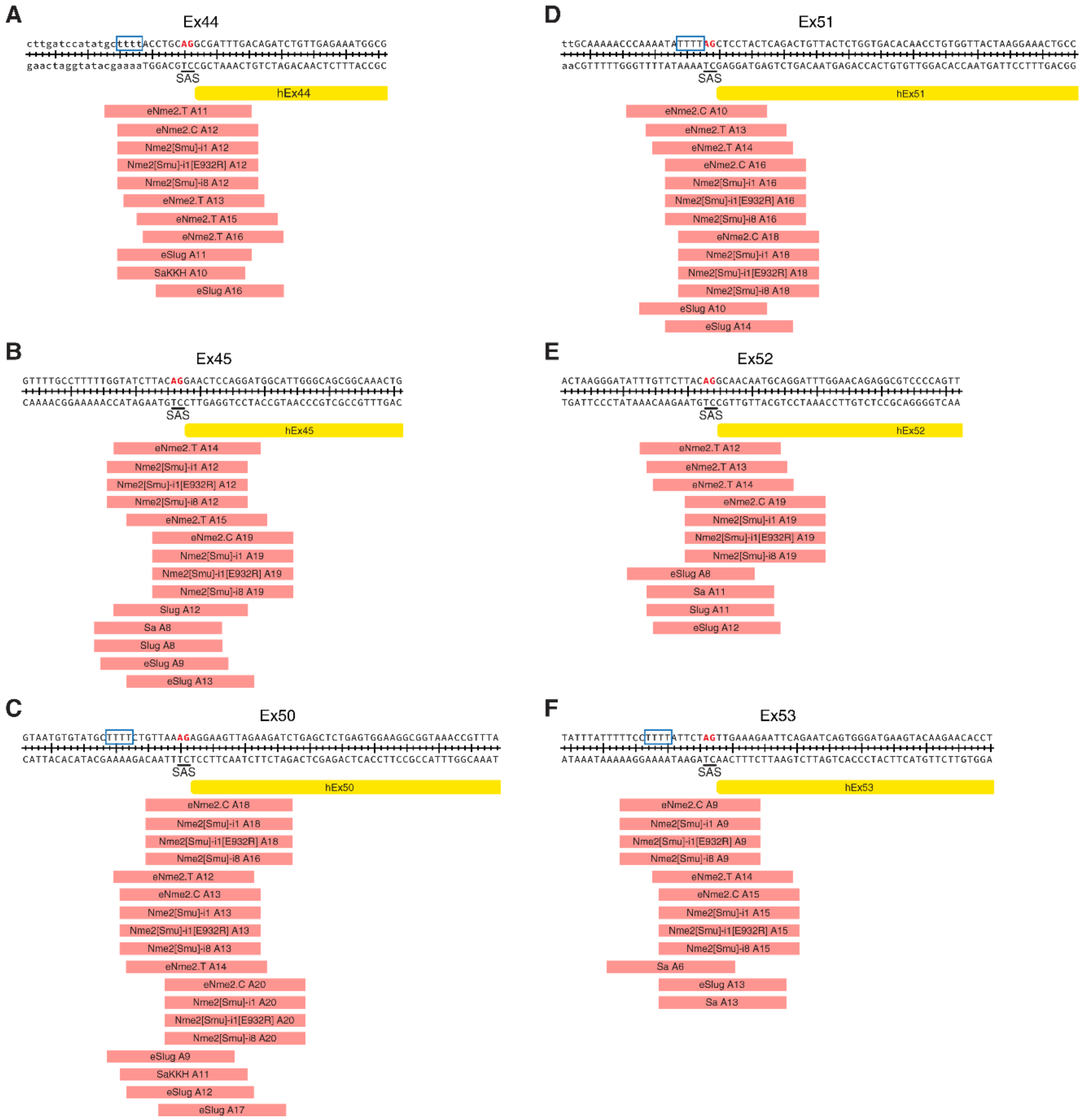

Supplemental Figure 2. Map of sgRNAs used in mini-ABE screening in HEK293T cells. sgRNAs targeting exon 44 (**A**), exon 45 (**B**), exon 50 (**C**), exon 51 (**D**), exon 52 (**E**), and exon 53 (**F**) splicing acceptor sites were labeled with their mini-ABE counterparts. Four T stretch sequences are highlighted in blue box.

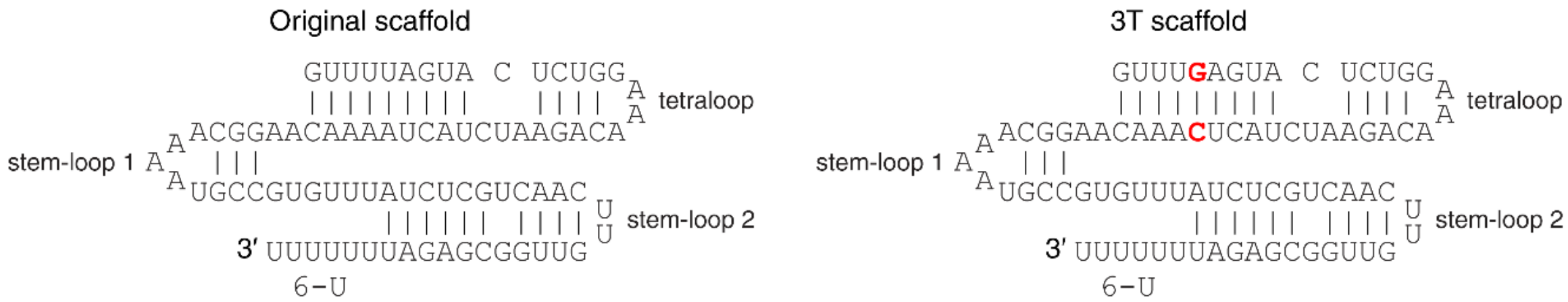

Supplemental Figure 3. Modified sgRNA scaffold for Sa-ABE8e and Slug-ABE8e. The 3T scaffold contained a T-to-G nucleotide change in the starting sequence to avoid premature transcriptional termination.

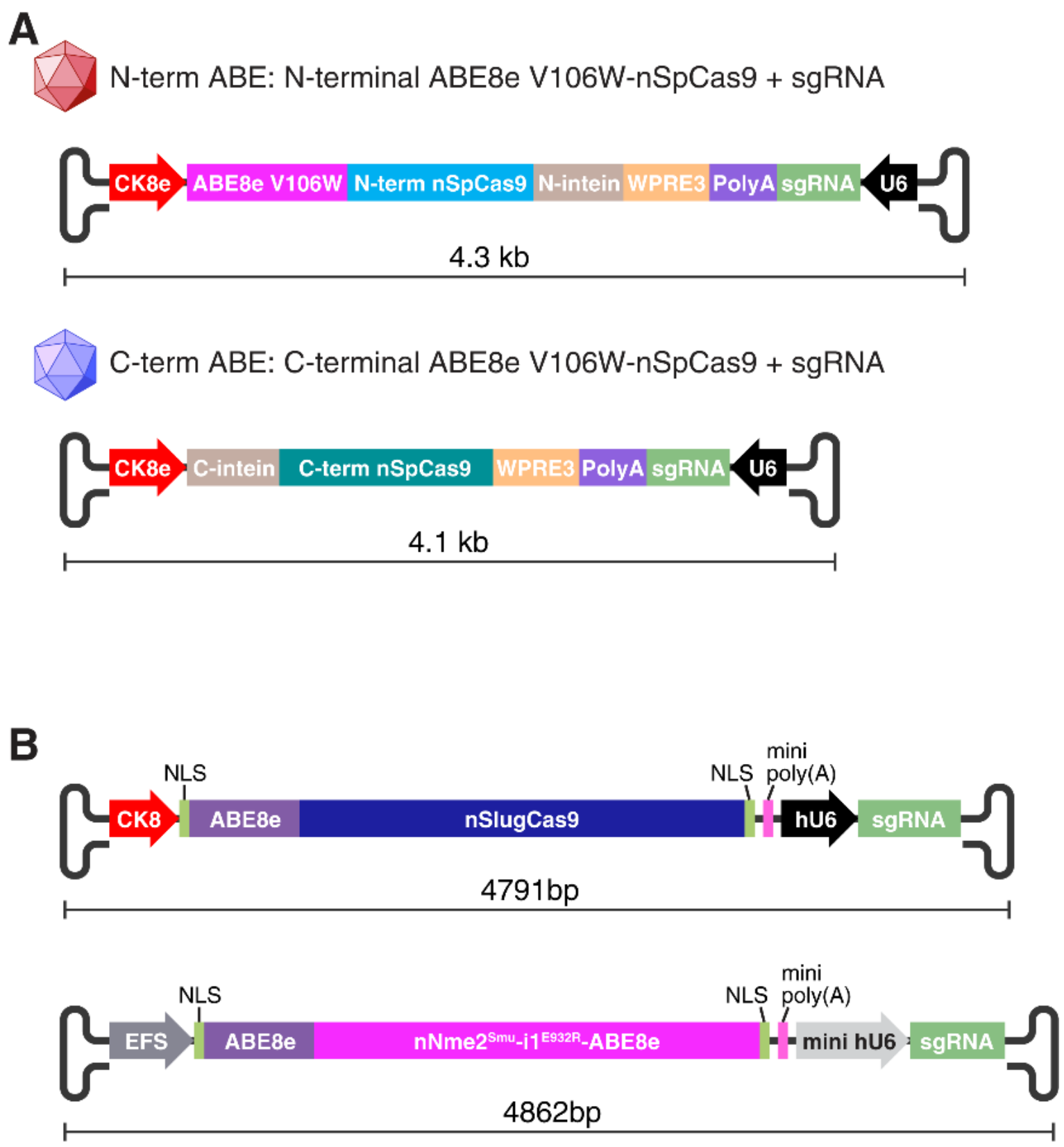

Supplemental Figure 4. Comparison of dual-AAV vectors with single AAV vectors. (**A**) Dual-AAV vectors were used in the previous study (1). Split-inteins were used to reconstitute Sp-ABE8e protein by trans-splicing. (**B**) Schematic of all-in-one AAV vectors. Both Slug-ABE8e (CK8 promoter and hU6 promoter) and Nme2.C-ABE8e (EFS promoter and mini hU6 promoter) constructs reached the AAV packaging limit (~4.8 kb).

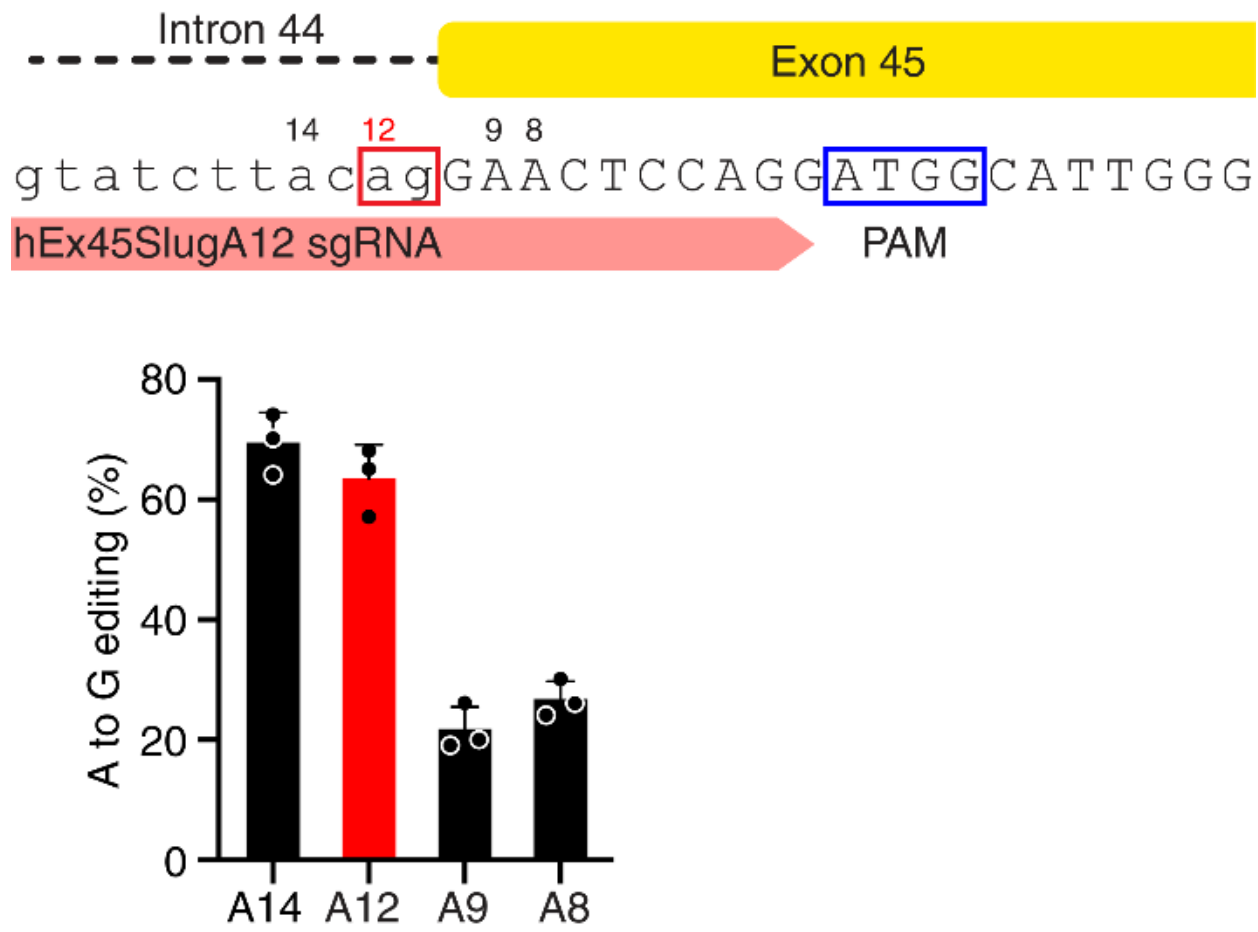

Supplemental Figure 5. Bystander editing in AAV6-SlugABE8e treated ∆Ex44 hiPSC-CMs. On-target A12 editing is shown in red. Bystander editing at A8, A9, and A14 is shown in black. Data are mean ± s.d. (n = 3 biologically independent replicates).

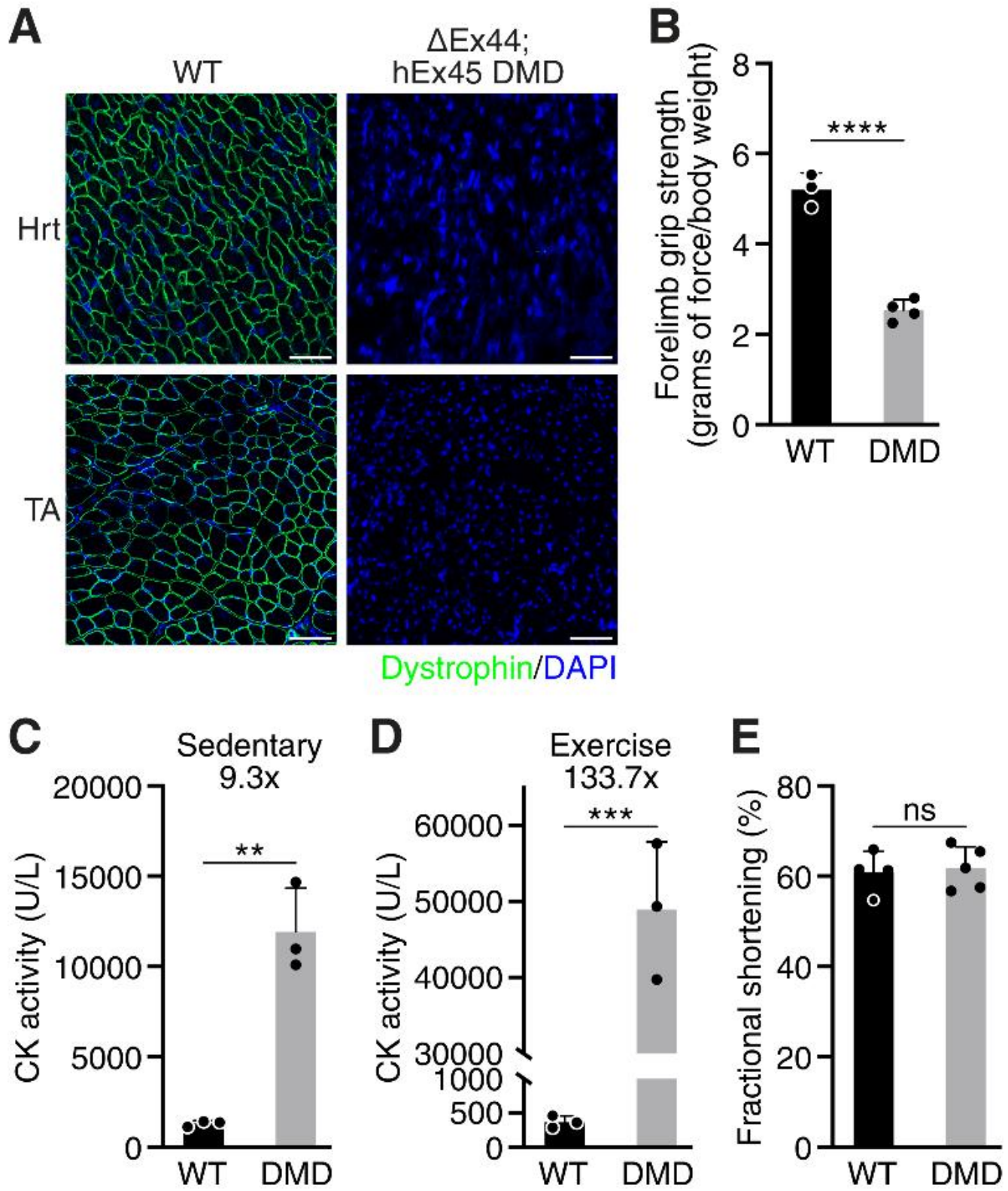

Supplemental Figure 6. Characterization of ΔEx44;hEx45 DMD mice. (**A**) Immunocytochemistry of dystrophin protein in heart and TA sections. Dystrophin positive fibers were observed in WT mice (left), while dystrophin protein is not expressed in ΔEx44;hEx45 mice (right). Dystrophin is in green; DAPI stains for nuclei in blue. Scale bar, 50 µm. (**B**) Forelimb grip strength of 4-week-old wild type (n=3) and ΔEx44;hEx45 mice (n=4). Grip strength was normalized to body weight (grams of force per gram of body weight). (**C**) Serum CK activity in 4-week-old WT (n=3) and ΔEx44;hEx45 mice (n=3) before (left) and (**D**) after exercise (after). (**E**) Echocardiography of 8-week-old wildtype mice (n=4) and ΔEx44;hEx45 mice (n=5) as measured by fractional shortening score. Data are mean ± s.d. ns = not significant, **p<0.01, ***p<0.001 by two-tailed, unpaired student t-test.

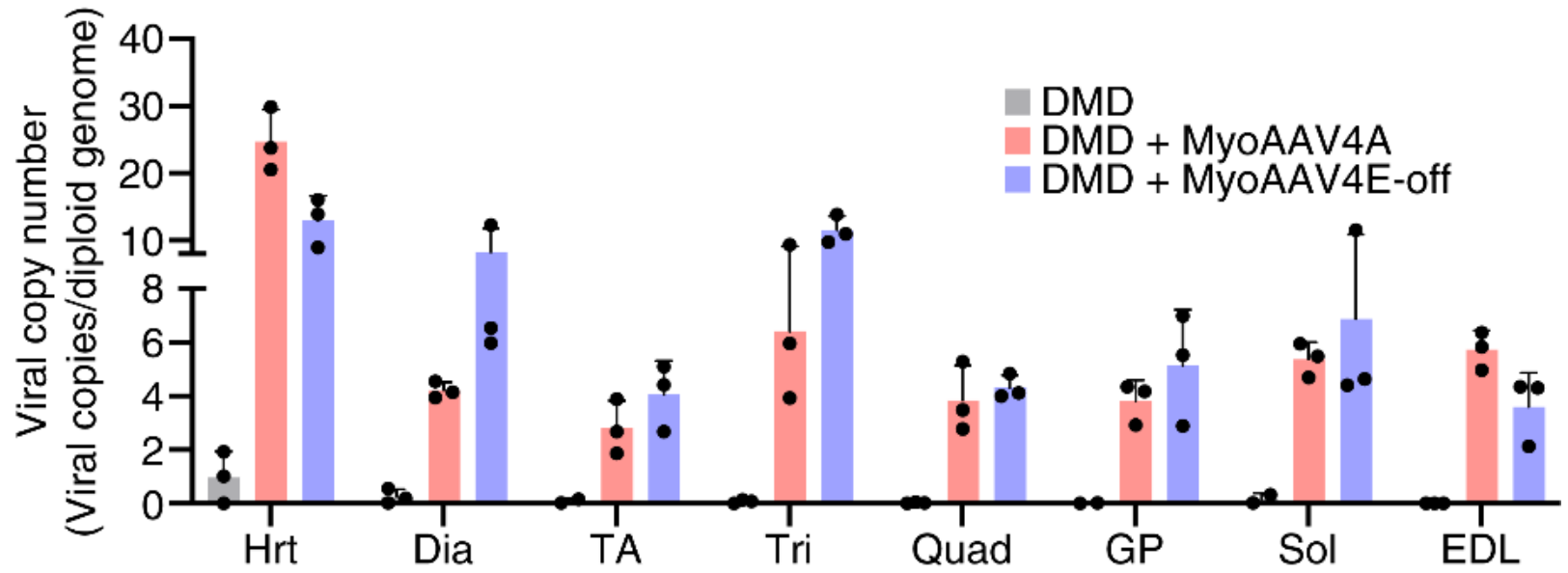

Supplemental Figure 7. Viral genome copy number in MyoAAV-treated ΔEx44;hEx45 DMD mice. Viral copy number of AAV from the heart (Hrt), diaphragm (Dia), tibialis anterior (TA), triceps (Tri), quadriceps (Quad), gastrocnemius and plantaris (GP), soleus (Sol), and extensor digitorum longus (EDL) with saline, MyoAAV4A, or MyoAAV4E-off treatment, normalized to mGusb by digital PCR. (n = 3 biologically independent replicates).

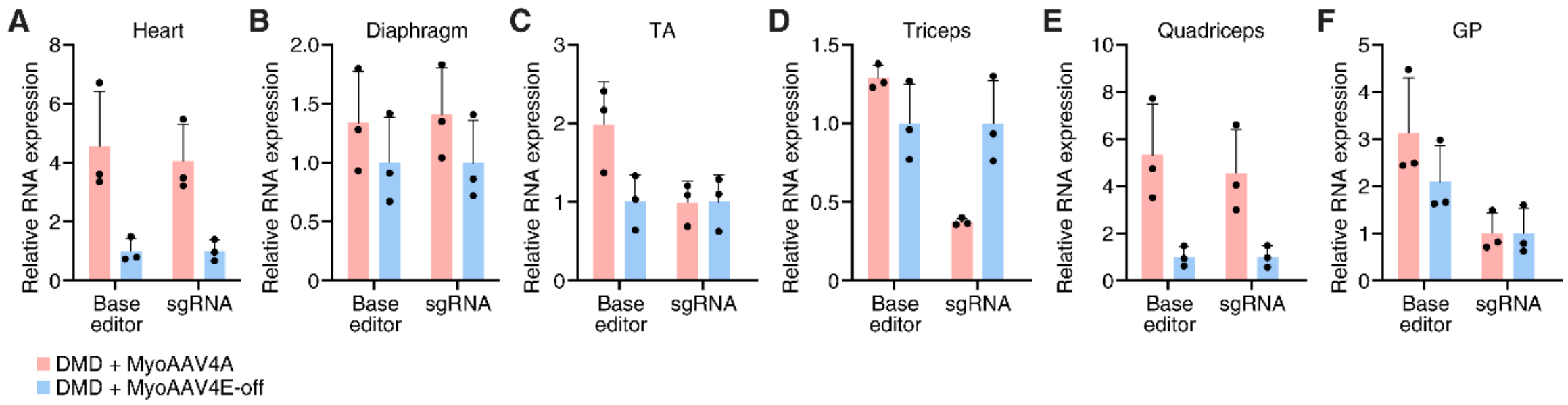

Supplemental Figure 8. Base editor and sgRNA expression in MyoAAV-treated ΔEx44;hEx45 DMD mice. qPCR analysis of base editor and sgRNA expression. Expression of the base editor and the sgRNA in MyoAAV4A-treated heart (**A**), diaphragm (**B**), tibialis anterior (TA) (**C**), triceps (**D**), quadriceps (**E**), and gastrocnemius-plantaris muscle (GP) (**F**) were normalized to MyoAAV4E-off treatment. (n = 3 biologically independent replicates).

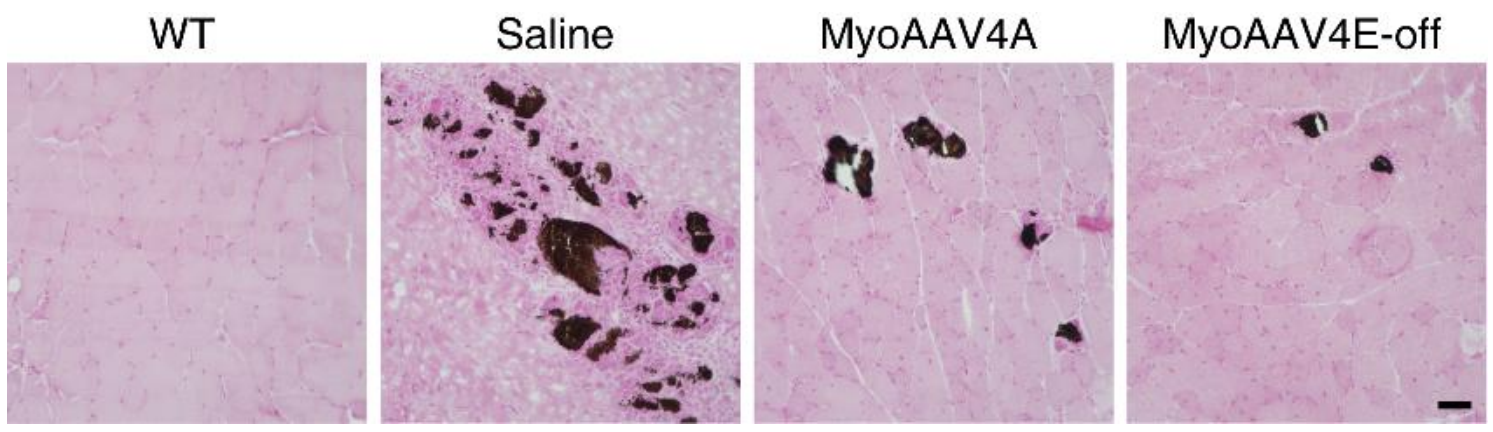

Supplemental Figure 9. Von Kossa staining of MyoAAV-treated ∆Ex44;hEx45 DMD mice. Visualization of calcification (dark) in triceps sections of ∆Ex44;hEx45 mice treated with saline, MyoAAV4A, or MyoAAV4E-off by Von Kossa staining. Scale bar, 100 µm. (n = 3 biologically independent replicates).

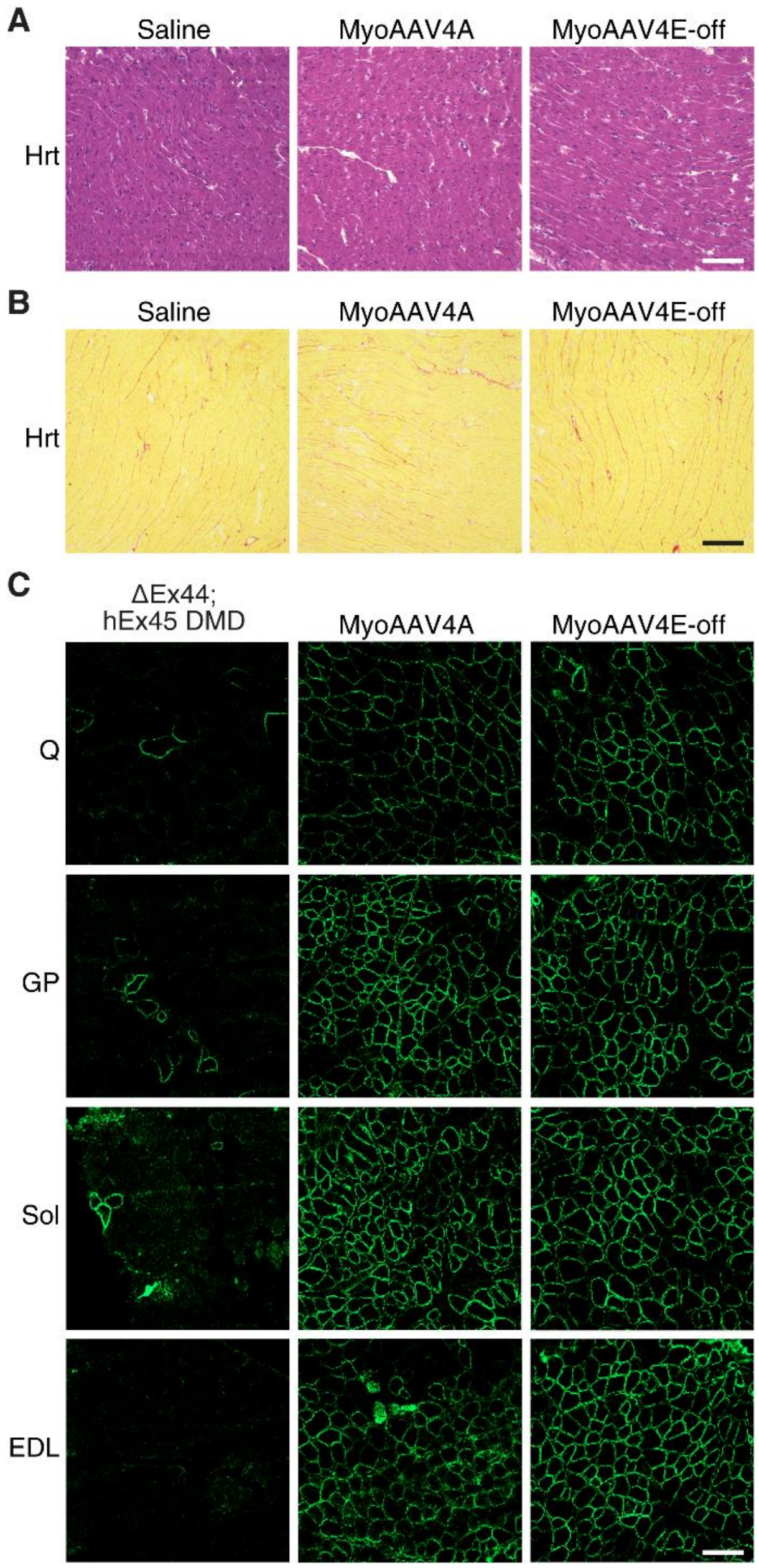

Supplemental Figure 10. Histological analysis of MyoAAV-treated ΔEx44;hEx45 DMD mice. (**A**) H&E staining of heart sections of ΔEx44;hEx45 mice treated with saline, MyoAAV4A, or MyoAAV4E-off. Scale bar, 200 μm. (**B**) Picrosirius red staining of heart sections ΔEx44;hEx45 mice treated with saline, MyoAAV4A, or MyoAAV4E-off. Scale bar, 200 μm. (**C**) **I**mmunohistochemistry of dystrophin in quadriceps (Q), gastrocnemius and plantaris (GP), soleus, and extensor digitorum longus (EDL) of ΔEx44;hEx45 mice treated with MyoAAV4A, or MyoAAV4E-off. Dystrophin is in green. Scale bar, 50 μm. (n = 3 biologically independent replicates).

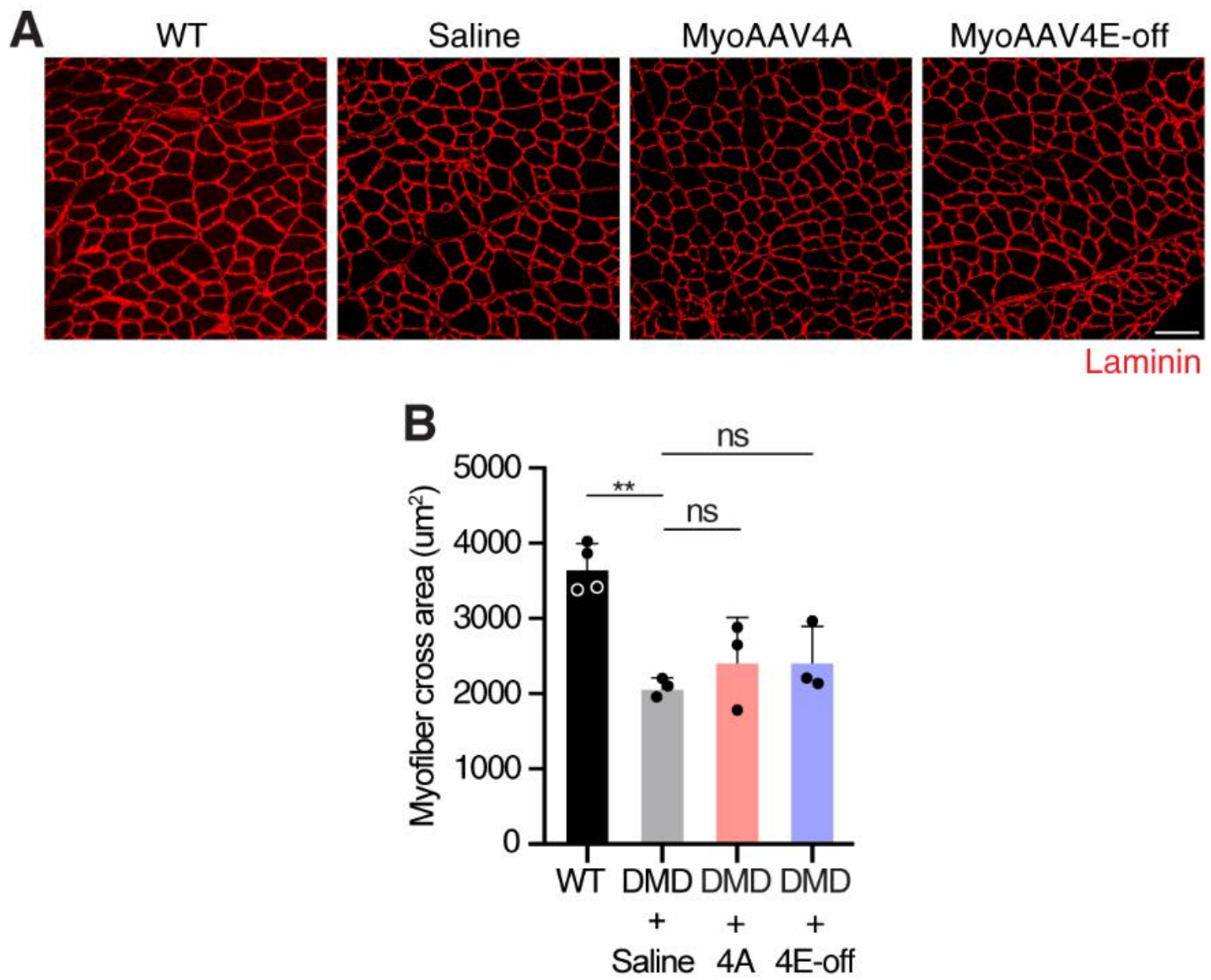

Supplemental Figure 11. Quantification of TA myofiber cross-sectional area in MyoAAV-treated ∆Ex44;hEx45 DMD mice. (**A**) Immunostaining of Laminin in ∆Ex44;hEx45 mice treated with saline, MyoAAV4A, or MyoAAV4E-off. Laminin is in red. (**B**) Quantification of myofiber cross area in ∆Ex44;hEx45 mice treated with saline, MyoAAV4A, or MyoAAV4E-off. Data are presented as mean ± SD. **P<0.01 by ordinary 1-way ANOVA. WT mice (n=4) and ∆Ex44;hEx45 mice treated with saline (n=3), MyoAAV4A (n=3), or MyoAAV4E-off (n=3). Scale bar, 100 µm.

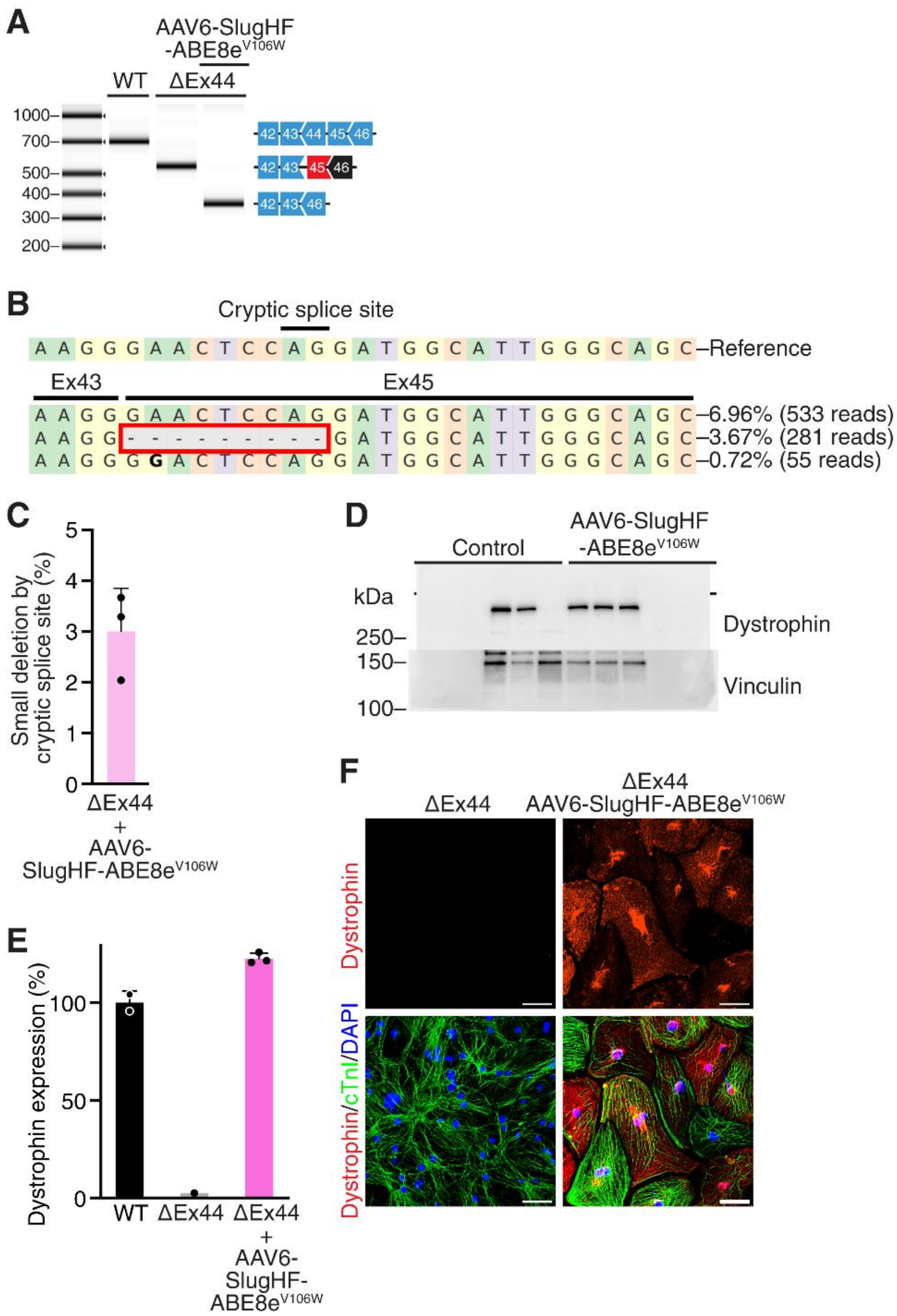

Supplemental Figure 12. All-in-one AAV6-SlugHF-ABE8e(V106W) induced beneficial exon skipping and restoration of Dystrophin expression in DMD ΔEx44 hiPSC-CMs. (**A**) RT-PCR analysis of AAV6-SlugHF-ABE8e(V106W) treated DMD ΔEx44 hiPSC-CMs. Corresponding exon structures are indicated on the right. (**B**) Deep amplicon sequencing of *DMD* RT-PCR products. A small deletion is marked in a red box. Boundaries of exon 43 and exon 45 and cryptic splice site “AG” are labeled. (**C**) Quantification of a small deletion by the cryptic splice site from deep amplicon sequencing (n=3). (**D**) Western blot showed that AAV6-SlugHF-ABE8e(V106W) treated DMD ΔEx44 hiPSC-CMs fully restored dystrophin expression. Vinculin is the loading control. (**E**)

Quantification of dystrophin expression of isogenic control, ∆Ex44, and AAV6-SlugHF-ABE8e(V106W) treated ∆Ex44 hiPSC-CMs. Relative intensity was measured as dystrophin expression normalized to vinculin expression compared to the isogenic control hiPSC-CMs. (**F**) Immunocytochemistry of AAV6-SlugHF-ABE8e(V106W) treated ∆Ex44 hiPSC-CMs (n=3). Dystrophin is in red; Cardiac troponin I (TnI) highlights CMs in green; DAPI stains for nuclei in blue. Scale bar, 50 µm. Data are mean ± s.d. (n = 3 biologically independent replicates). For RT-PCR (**A**), WT (n=1), ∆Ex44 (n=1), and ∆Ex44+AAV6 (n=1). For western blot (**D**-**E**), WT (n=2), ∆Ex44 (n=1), and ∆Ex44+AAV6 (n=3).).

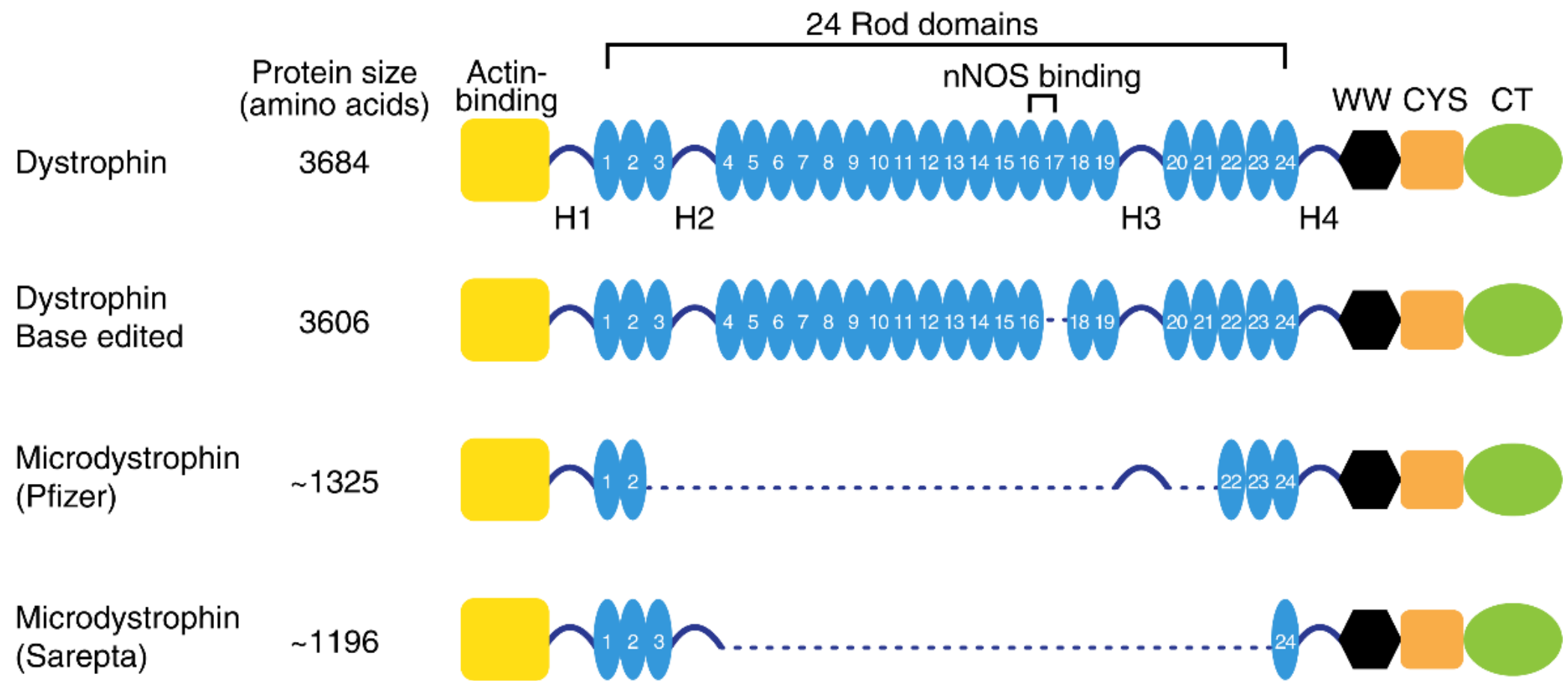

Supplemental Figure 13. Comparison of gene edited dystrophin and micro-dystrophin. Full-length dystrophin protein contains the actin-binding domain, rod domains, WW domain, cysteine-rich domain, and C-terminal domain. Base editing-mediated exon 45 skipped dystrophin contains more domains than micro-dystrophin.

Small Cas9s	Size	PAM
Cje1Cas9	984 aa	NNNVRYAC
Cje2Cas9	987 aa	NNNNCYWT
Cje3Cas9	1000 aa	NNNNCYA
SaCas9	1053 aa	NNGRRT
SaKKH-Cas9	1053 aa	NNHRRT
SlugCas9	1055 aa	NNGG
PpCas9	1056 aa	NNNNRTT
Cje2Cas9	1061 aa	NNGG
Cje3Cas9	1082 aa	NNNNGTAA
SaCas9	1082 aa	NNNNCC
SaKKH-Cas9	1081 aa	NNNNCAAA
SlugCas9	1121 aa	NNRGAA
PpCas9	1130 aa	NNGYRA

Supplemental Table 1. List of smaller Cas9 variants. Cas9 variants are ordered by their protein size with their PAM sequences. (R: A or G; Y: C or T; W: A or T; H: A or C or T; V: A or C or G)

	Red text indicates MiSeq adapter sequence for amplicon deep sequencing.
Deep Amplicon Sequencing (DAS) hE45SlugA12 ON F	TCGTCGGCAGCGTCAGATGTGTATAAGAGACAGCTTTCT TTGCCAGTACAACTGC
DAS hE45SlugA12 ON R	GTCTCGTGGGCTCGGAGATGTGTATAAGAGACAGATCTG TCGCCCTACCTCTT
DAS hE45SlugA12 OFF1 F	TCGTCGGCAGCGTCAGATGTGTATAAGAGACAGGCCACC ATGCCCAGATAAA
DAS hE45SlugA12 OFF1 R	GTCTCGTGGGCTCGGAGATGTGTATAAGAGACAGTGCAA TGCATGATCCTTAAGTAGA
DAS hE45SlugA12 OFF2 F	TCGTCGGCAGCGTCAGATGTGTATAAGAGACAGCAACCA GAATAGGAAGGGATGT
DAS hE45SlugA12 OFF2 R	GTCTCGTGGGCTCGGAGATGTGTATAAGAGACAGCACAT GTACAGGTCAGGATGTAG
DAS hE45SlugA12 OFF3 F	TCGTCGGCAGCGTCAGATGTGTATAAGAGACAGGCCAAT ATAAATATTCTGTACTGGC
DAS hE45SlugA12 OFF3 R	GTCTCGTGGGCTCGGAGATGTGTATAAGAGACAGCTTAG TGGCTAACACCTTAAC
DAS hE45SlugA12 OFF4 F	TCGTCGGCAGCGTCAGATGTGTATAAGAGACAGGTCTTT AGCAAGACAAAATGCC
DAS hE45SlugA12 OFF4 R	GTCTCGTGGGCTCGGAGATGTGTATAAGAGACAGCCCTT TATTAGGAAAAGGAAGTTCC
DAS hE45SlugA12 OFF5 F	TCGTCGGCAGCGTCAGATGTGTATAAGAGACAGCCAAAC CACCTGTAAGGTATGA
DAS hE45SlugA12 OFF5 R	GTCTCGTGGGCTCGGAGATGTGTATAAGAGACAGT GTTGGAAGGTGACCCTAAT
DAS hE45SlugA12 OFF6 F	TCGTCGGCAGCGTCAGATGTGTATAAGAGACAGAC ATCAGCAGTCTCCTTTCC
DAS hE45SlugA12 OFF6 R	GTCTCGTGGGCTCGGAGATGTGTATAAGAGACAGGCCTA CTGGGTTCCTTCTTC
DAS hE45SlugA12 OFF7 F	TCGTCGGCAGCGTCAGATGTGTATAAGAGACAGCCTCCA CCTACTTAACCTCATTC
DAS hE45SlugA12 OFF7 R	GTCTCGTGGGCTCGGAGATGTGTATAAGAGACAGCAGGC TTGCTACTGCTAACT

DAS hE45SlugA12 OFF8 F	TCGTCGGCAGCGTCAGATGTGTATAAGAGACAGGCAGTT GTCTGCACTGTAATG
DAS hE45SlugA12 OFF8 R	GTCTCGTGGGCTCGGAGATGTGTATAAGAGACAGCATGA TGGTGCATGTCTGTAATC
DAS hE45SlugA12 OFF9 F	TCGTCGGCAGCGTCAGATGTGTATAAGAGACAGAGCAAA GGAGAAAGAGGGATTT
DAS hE45SlugA12 OFF9 R	GTCTCGTGGGCTCGGAGATGTGTATAAGAGACAGACTCC CAGGACTGTCGTATT
DAS hE45SlugA12 OFF10 F	TCGTCGGCAGCGTCAGATGTGTATAAGAGACAGCC CTTTCAGTCCTTAGCCATAC
DAS hE45SlugA12 OFF10 R	GTCTCGTGGGCTCGGAGATGTGTATAAGAGACAGC TCCCTCTTTCTCCACCTACT
DAS hE45SlugA12 OFF11 F	TCGTCGGCAGCGTCAGATGTGTATAAGAGACAGGG CATGGCTTGAATTACATGAG
DAS hE45SlugA12 OFF11 R	GTCTCGTGGGCTCGGAGATGTGTATAAGAGACAGG TTGTCTGACATCCACCATCT
DAS hE45SlugA12 OFF12 F	TCGTCGGCAGCGTCAGATGTGTATAAGAGACAGCA ACCACTTCAGTTCGGAGTTAG
DAS hE45SlugA12 OFF12 R	GTCTCGTGGGCTCGGAGATGTGTATAAGAGACAGG CATACAGGTGTGACAGCATAG
DAS hE45SlugA12 OFF13 F	TCGTCGGCAGCGTCAGATGTGTATAAGAGACAGCC GGCTTATCCTTGCTCTTTA
DAS hE45SlugA12 OFF13 R	GTCTCGTGGGCTCGGAGATGTGTATAAGAGACAGC ATCTCTTGACCTCGTGATCTG
DAS hE45SlugA12 OFF14 F	TCGTCGGCAGCGTCAGATGTGTATAAGAGACAGGC GACTGAGTCACCTATACAAC
DAS hE45SlugA12 OFF14 R	GTCTCGTGGGCTCGGAGATGTGTATAAGAGACAGG ATCTGCATCAGCCTCATCTT
DAS hE45SlugA12 OFF15 F	TCGTCGGCAGCGTCAGATGTGTATAAGAGACAGTT GCTGCTTCCTGTCTTCTC
DAS hE45SlugA12 OFF15 R	GTCTCGTGGGCTCGGAGATGTGTATAAGAGACAGC CTCATTTCCAACCCTGACTATTA
DAS hE45SlugA12 OFF16 F	TCGTCGGCAGCGTCAGATGTGTATAAGAGACAGGG CACAACAAACCTTGCATTA

DAS hE45SlugA12 OFF16 R	GTCTCGTGGGCTCGGAGATGTGTATAAGAGACAGCCTTTAGGTGGGAATCACAGTAG
DAS hE45SlugA12 OFF17 F	TCGTCGGCAGCGTCAGATGTGTATAAGAGACAGCACCACCATCTCAGGTAAGAATAA
DAS hE45SlugA12 OFF17 R	GTCTCGTGGGCTCGGAGATGTGTATAAGAGACAGGGGTGTGAGGAACAAAGGAA
DAS hE45SlugA12 OFF18 F	TCGTCGGCAGCGTCAGATGTGTATAAGAGACAGGGTTCTCGTTGCCACATTTC
DAS hE45SlugA12 OFF18 R	GTCTCGTGGGCTCGGAGATGTGTATAAGAGACAGTCCCAAAGTGCTGGGATTATAG
DAS hE45SlugA12 OFF19 F	TCGTCGGCAGCGTCAGATGTGTATAAGAGACAGCTGGCAACAGACAGCATGA
DAS hE45SlugA12 OFF19 R	GTCTCGTGGGCTCGGAGATGTGTATAAGAGACAGGTGGACACTCGGTAAGCATTAG
DAS hE45SlugA12 OFF20 F	TCGTCGGCAGCGTCAGATGTGTATAAGAGACAGGGATGATCTTTCCTGTCCTCAC
DAS hE45SlugA12 OFF20 R	GTCTCGTGGGCTCGGAGATGTGTATAAGAGACAGACCATGTACCTCAGTTGGAAAT
DAS hE45SlugA12 OFF21 F	TCGTCGGCAGCGTCAGATGTGTATAAGAGACAGTCTCTCTCCTCAGCAGGAATAA
DAS hE45SlugA12 OFF21 R	GTCTCGTGGGCTCGGAGATGTGTATAAGAGACAGTGGCTACTAGCGGACACATA
DAS hE45SlugA12 mE43 *Dmd* F	TCGTCGGCAGCGTCAGATGTGTATAAGAGACAGAGGTGAAAGTACAGGAAGCCGT
DAS hE45SlugA12 mE46 *Dmd* R	GTCTCGTGGGCTCGGAGATGTGTATAAGAGACAGCTGCTGCTCATCTCCAAGTGGA
hE45SlugA12 hE42 *Dmd* F	GCCCTATTAGAAGTGGAACAAC
hE45SlugA12 hE46 *Dmd* R	GGTTCAAGTGGGATACTAGC
DAS hE45SlugA12 hE43 *Dmd* F	TCGTCGGCAGCGTCAGATGTGTATAAGAGACAGGAAAGGGTGAAGCTACAGGAA
DAS hE45SlugA12 hE46 *Dmd* R	GTCTCGTGGGCTCGGAGATGTGTATAAGAGACAGCTGCTGCTCATCTCCAAGTGGA
dPCR AAV ITR F	GGAACCCCTAGTGATGGAGTT
dPCR AAV ITR R	CGGCCTCAGTGAGCGA

dPCR AAV ITR Probe	/56-FAM/CACTCCCTC/ZEN/TCTGCGCGCTCG/3IABkFQ/
NheI-Slug-HiFi-F	AGACACTCAGGAGCCAGCCAGCGCTA
ABE8eV106W-HiFi-R	TTTGAGTTCCTCCATCCAAACACCACGCG
ABE8eV106W-HiFi-F	CGCGTGGTGTTTGGATGGAGGAACTCAAA
R247A-SlugHF-HiFiR	GTACTTGACGGAAGCCAGTTCGTCGG
R247A-SlugHF-HiFi-F	CCGACGAACTGGCTTCCGTCAAGTAC
N415A-SlugHF-HiFi-R	AGCGAAGATTTCCATTTGAGCGCGTGAGGAGTAC
T421A-SlugHF-HiFi-F	GCTCAAATGGAAATCTTCGCTCACCTGAACATC
R656A-SlugHF-HiFi-R	ATTTGTCAATTCAGCGGTGGCATAGCGG
R656A-SlugHF-HiFi-F	CCGCTATGCCACCGCTGAATTGACAAAT
BlpI-SlugHF-HiFi-R	GAACCTGTAGGGCTTAATGCTGAGCTTG
Sanger Sequencing (SS) *DMD* hEx44 F	GCAGGAAACTATCAGAGTG
SS *DMD* hEx44 R	ACACCTTGCTGTTACGAT
SS *DMD* hEx45 F	GTCTTTCTGTCTTGTATCCTTTGG
SS *DMD* hEx45 R	AATGTTAGTGCCTTTCACCC
SS *DMD* hEx50 F	TGCCTGGAGAAAGGGTTT
SS *DMD* hEx50 R	GCACAGTCAATAACACAAAGGT
SS *DMD* hEx51 F	AAGTTACTTGTCCAGGCATGAGAAT
SS *DMD* hEx51 R	GCTGAGAGAGAAACAGTTGCCTAAG
SS *DMD* hEx52 F	TTCTTACTCAAGGCATTCAGAC
SS *DMD* hEx52 R	GGTCACCACACCCATCAAT
SS *DMD* hEx53 F	GGGAAATCAGGCTGATGGGT
SS *DMD* hEx53 R	GTCTACTGTTCATTTCAGC
Gapdh-DNA-F	CATTCAGGTCTCTGGGTCCTGGATG
Gapdh-DNA-R	GATGGTTCCCAGGATAGGACTCAGG
Gapdh-DNA-probe	TGGTGCACACTCCAAGGACTCCTCGT
Gusb-cDNA-F	CTGCAGGGTTTCGAGCAGCAAT
Gusb-cDNA-R	GGGCTGCTTCTTGGGTGATGTC
Gusb-cDNA-probe	TCGGGCCCAGTCTTGGACATGCCT
ABE-Kozak-NLS-F	GCCACCATGAAACGGACA
ABE-SV40-NLS-R	GACTTTCCGCTTCTTCTTTGG
ABE-NLS-probe	CGACGGAAGCGAGTTCGAGTCA

sgRNA-Slug-hE45-F	CTTACAGGAACTCCAGGGTTTG
sgRNA-Slug-Scaffold-R	CGCCAACAAGTTGACGAGATA
sgRNA-Slug-Scaffold-probe	TCTGGAAACAGAATCTACTCAAACAAGGCA
Rplp0 qF	ATCCCTGACGCACCGCCGTGA
Rplp0 qR	TGCATCTGCTTGGAGCCCACGT
Tnf qF	KiCqStart SYBR Green Primers (Millipore Sigma)
Tnf qR	KiCqStart SYBR Green Primers (Millipore Sigma)
Ifng qF	KiCqStart SYBR Green Primers (Millipore Sigma)
Ifng qR	KiCqStart SYBR Green Primers (Millipore Sigma)

Supplemental Table 2. List of primers used in this study

1. Chai AC, et al. Single-swap editing for the correction of common Duchenne muscular dystrophy mutations. *Molecular Therapy Nucleic Acids*. 2023;32:522-535. doi:10.1016/j.omtn.2023.04.009
